# Cutting Edge Methods for Non-Invasive Disease Diagnosis Using E-Tongue and E-Nose Devices

**DOI:** 10.3390/bios7040059

**Published:** 2017-12-07

**Authors:** Jessica Fitzgerald, Hicham Fenniri

**Affiliations:** Department of Chemical Engineering, Northeastern University, 313 Snell Engineering Center, 360 Huntington Avenue, Boston, MA 02115, USA; fitzgerald.je@husky.neu.edu

**Keywords:** electronic nose, electronic tongue, biomimetic sensors, cross-reactivity, volatile organic compounds, metabolomics, diagnostics

## Abstract

Biomimetic cross-reactive sensor arrays (B-CRSAs) have been used to detect and diagnose a wide variety of diseases including metabolic disorders, mental health diseases, and cancer by analyzing both vapor and liquid patient samples. Technological advancements over the past decade have made these systems selective, sensitive, and affordable. To date, devices for non-invasive and accurate disease diagnosis have seen rapid improvement, suggesting a feasible alternative to current standards for medical diagnostics. This review provides an overview of the most recent B-CRSAs for diagnostics (also referred to electronic noses and tongues in the literature) and an outlook for future technological development.

## 1. Introduction

### 1.1. Current Diagnostic Methods and Motivation

In the last few decades, research in the medical field has provided tools to help doctors reach medical diagnoses. While these tools have been successfully used and implemented to detect and identify certain diseases, many conditions are diagnosed using methods that can be costly, painful to the patient, and/or inaccurate, especially in the early stages of their development [1,2]. Thus, there remains a need for the devices that target these facets to provide early diagnosis and analysis for each patient that is altogether more cost effective, accurate, and non-invasive.

For example, cancer detection and treatment remain significant challenges that affect many Americans. In 2017 alone, the American Cancer Society estimates that there were 222,500 new diagnoses of lung cancer, and 155,870 deaths due to lung cancer [3]. Though it is not as prevalent as lung cancer, colorectal cancer is the third leading type of cancer, with a projected 135,430 individuals newly diagnosed in the United States (U.S.) in 2017 and carrying a high mortality rate of 17.7% for men and 12.4% for women [4]. Furthermore, colorectal cancer diagnosis requires a colonoscopy and polyp biopsy, which is both invasive and costly. For these and all other types of cancer, treatment and maintenance costs are high. In 2014, the national cost of cancer was estimated to be $87.8 billion [5]. Recent research has shown, however, that early diagnosis is a key factor for cancer survival and treatment cost, with the five year survival rate increasing for local, regional, and distant stage disease, and the overall cost of cancer is decreasing for those treated in the early stages [6]. 

For chronic airway diseases, such as chronic obstructive pulmonary disease (COPD) and obstructive sleep apnea (OSA), early diagnosis is imperative to improve quality of life and enact preventative measures to reduce adverse health effects and comorbid pathologies. In 2017, the COPD Foundation estimated that 6.3% (20.3 million) of U.S. citizens had been diagnosed with COPD, and in most cases, diagnosis did not take place until 50% of lung function was lost to the disease [5]. Regarding OSA, studies from 2008 to 2013 reported a mean prevalence of OSA of 37% in men and 50% in women, indicating a drastic increase from studies performed during the 1990s and early 2000s. Related to age and obesity, OSA significantly increases the risk of early death and cardiovascular diseases, including strokes [7]. Furthermore, OSA diagnosis methods are lengthy and uncomfortable for the subject, requiring an in-patient sleep study with expensive and specialized equipment. 

Concerning the accuracy of diagnosis, there remains a need for analytical tools for patients with neurodegenerative diseases, such as Alzheimer’s Disease and Parkinson’s Disease, and mental illnesses resulting from chronic stress and anxiety. Mental illness is a widespread issue in the United States. In 2015, the National Institutes of Health (NIH) reported that 43.4 million adults (17.9%) suffered from a mental illness [5]. In 2013, costs associated with mental health services totaled $201 billion, topping the list of the most costly conditions [8]. The cause of many of these illnesses remains unknown. Treatment is administered on a trial-and-error basis, which reduces effectiveness and raises the risk of serious side effects. 

Finally, pathologies that are considered metabolic disorders, such as diabetes and inflammatory bowel disease (IBD) including Crohn’s disease (CD) and ulcerative colitis (UC), and chronic kidney disease would benefit greatly from a device that can not only diagnose the disease, but can also monitor disease progression. The National Diabetes Statistics Report estimated that 29.1 million people (9.3%) in the U.S. have some form of diabetes, with 27.8% of those with diabetes still undiagnosed and untreated, increasing the risk of adverse health effects [9]. For IBD, incidence rates have increased steadily over time worldwide, with highest incidence in Europe and the USA (2.5 million and >1 million affected, respectively) [10]. Moreover, it was reported that of the countries studied, the U.S. had the highest rate of hospitalizations for IBD-related illness [11].

Over the past decade, advances in molecular technologies have demonstrated that identification of cellular changes at the molecular level may be a promising approach to early disease detection [12]. Further, being able to measure cellular changes at an early phase of the development of diseases may enable the identification of the pathophysiological processes underlying these conditions, thus informing treatment development. An accurate device that can provide a unique patient profile would contribute significantly to the development of a specific treatment method based on the individual’s needs, and could act as a tool to understanding the physiological processes that are involved for each illness.

### 1.2. “Omics” Profiling and Impact on Personalized Medicine

Technological advances have made it possible to produce highly detailed, patient-specific molecular profiles of both vapor and liquid samples. By using various “omics” methods including proteomics, genomics, and metabolomics, researchers and health care professionals have been able to associate specific biomarkers, i.e., genes, proteins, and molecules with patients’ diseases. Once identified, these biomarkers serve as reliable indicators for the most beneficial personalized targeted therapies for the patient. This, in turn, has the potential to improve quality of life, treatment effectiveness, and reduce undesirable secondary effects and associated treatment costs. Both the National Institutes of Health (NIH) and the Food and Drug Administration (FDA) have realized the benefit of a personalized medicine approach. In an article published by the New England Journal of Medicine, authors M. A. Hamburg and F. S. Collins stressed “the success of personalized medicine depends on having accurate diagnostic tests that identify patients who can benefit from targeted therapies” [13]. 

As diagnostic device development trends toward enhanced portability, ease-of-use, throughput, and affordability, personalized medicine via patient-specific disease profiling is now a very realizable goal [14,15]. This review article aims to highlight the potential of devices that employ biomimetic cross reactive sensor arrays (B-CRSAs) to provide non-invasive disease diagnosis, and to help advance personalized medicine through biomarker identification. The subsequent sections describe the most recent methods in B-CRSA diagnostics for a wide variety of pathologies including cancer, airway diseases, mental and neurological diseases, and metabolic disorders, explore cutting-edge sensor technology for these and other diseases, and outline future directions in device development.

## 2. Electronic Noses and Tongue Devices Background Technology

### 2.1. Mammalian Olfactory System as a Platform for Cross-Reactive Sensing

Over the past several decades, many research efforts have looked to the mammalian olfactory system as a model for the development of a medical diagnostic device that meets the necessary requirements. Overall, there are about 1000 genes that encode olfactory receptors (ORs), and each OR has multiple sites for odorant binding, enabling detection of more than one odorant for each OR—a characteristic called cross-reactivity [16,17]. Each combination of activated receptors creates a unique signaling code for a specific odorant, making it possible to distinguish between thousands of odorants [17]. In March of 2015, The Guardian newspaper published an article about a dog, named Frankie, who could detect thyroid cancer with 88% accuracy among patients. Thyroid cancer is notoriously difficult to detect by conventional methods, and it is hard to tell if the entire tumor has been removed post-surgery. Frankie, however, was trained to lie down after smelling patients’ urine samples if he detected metastatic cancer [18].

Inspired by the sensing mechanism of the mammalian olfactory system, medical devices known as “electronic/artificial noses or tongues” (e-noses, e-tongues), containing biomimetic cross-reactive sensor arrays (B-CRSAs) have been developed to detect specific olfactory (smell) and gustatory (taste) elements present in both vapor and liquid samples, respectively. The analyte mixtures represent specific pathological profiles, and these B-CRSAs have proven to be successful at diagnosing a wide variety of pathologies, providing cost-effective, minimally invasive, and highly accurate analyte profile analysis and classification.

The sensors employed in e-Noses fall into three general categories: gravimetric, electrical, and optical, allowing for characterization of analytes based on mass, electrical properties (e.g., conductance or impedance), and electron/photon interactive properties, respectively. Gravimetric sensors are either piezoelectric crystals or microcantilevers, which resonate at a specific frequency. Upon binding with an analyte, the resonant frequency of the sensor drops in proportion with the added mass. Electrical e-noses consist of an electronic circuit that is connected to a network of sensory materials—most commonly to conductive polymers or metal oxides—that provide an electrical response upon binding with a specific known analyte. This response is characterized by monitoring sensor conductivity, resistivity, or voltage change during analyte exposure. Finally, optical sensors work by displaying a shift in emission or absorption of different types of electromagnetic radiations upon binding with a desired analyte. There are two popular means of detection: fluorescent sensors, which fluoresce upon analyte binding, and colorimetric sensors, which display a visible color change upon analyte binding.

### 2.2. Disease Diagnoses with B-CRSAs

B-CRSAs have great potential to advance personalized medicine. While the devices have been used in a wide variety of applications from consumer goods analysis [19,20,21,22,23,24,25] to explosives detection [26,27,28,29], some of the most pioneering work has been done in medical diagnostics via vapor and liquid samples from subjects. Many disease pathologies have been identified from the unique combination of metabolites, or metabolic by-products, produced [15,30,31,32,33,34,35,36,37,38,39,40,41]. Some of these metabolites are volatile organic compounds (VOCs), which are small molecules that enter the exhaled breath through gas exchange at the alveolar-capillary membrane of the respiratory tract [42], or can even be found in body odor [43,44] or the vapor head space of bacterial [45,46,47], urine [31,33,45,48], or fecal samples [49,50,51]. Others are protein or molecular biomarkers found in liquid samples such as sweat [34,52,53,54], urine [35,55,56], and saliva [30,52,57,58,59,60,61,62]. While the metabolites produced from each disease are thought to be primarily from oxidative stress, the subsequent effect of each disease on the body is unique and leads to the production of disease-specific metabolomic profiles [37,63].

**Figure 1:**
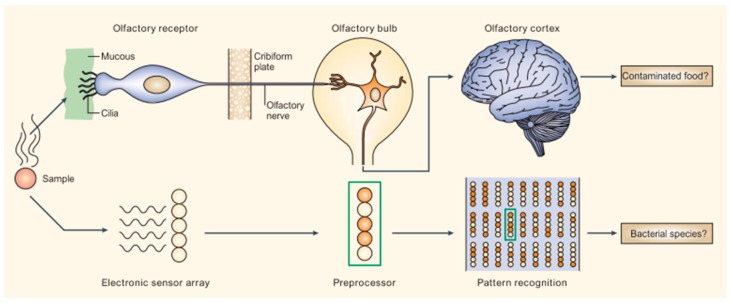
Parallels between mammalian olfactory system and electronic nose and tongue devices. Used with permission from [64].

E-noses and e-tongues consist of a CRSA, which is capable of interacting with multiple vapor or liquid analytes, a signal transduction mechanism, and pattern recognition software to classify the samples in question (Figure 1) [64]. B-CRSAs are able to differentiate diseases via “fingerprint” (FP) outputs; that is, each patient’s metabolite profile produces a unique response pattern from the sensor array, enabling disease differentiation by comparing new patient FPs to two controls: FPs of healthy patients and FPs of patients for whom a disease diagnosis has been confirmed. B-CRSAs are exposed to the collected samples, and multivariate data analysis—such as principle component analysis (PCA) or neural network analysis—is used for pattern recognition and clustering to classify each FP.

The development of more extensive and selective sensing mechanisms alongside advances in electronics and signal processing, B-CRSA devices have become smaller, more selective, and more sensitive, with fast data processing time and facile readout of vapor analysis [65,66,67]. Recent advances in research and technology have progressed toward personalized medicine, where large amounts of data can be analyzed to identify specific biomarkers of a disease in each individual patient. When used in parallel with methods such as GC-MS and NMR-metabolomics [38,68], a comprehensive breathomics approach can be successfully developed for disease diagnoses. Such a device could offer clinically relevant opportunities for objective tracking of symptoms, changes over time, response to treatment, as well as resilience to future environmental stressors. Overall, e-nose and e-tongue devices offer promise as a novel area to develop as they offer a potentially highly impactful solution for early detection of a range of medical and psychiatric diagnoses, as well as the potential for more closely matching treatment to patient-specific disease pathology and monitoring of treatment response.

## 3. Cancer Metabolomics

### 3.1. Lung Cancer

Several studies have been performed that demonstrate the success of lung cancer identification through e-nose analysis of exhaled breath [32]. Indeed, airways diseases including lung cancer were some of the first to be identified via profiling of VOCs in exhaled breath. One successful clinical trial performed by Santonico et al. proposed a more consistent breath sampling technique via endoscopic probe for the improved discrimination of lung cancer-specific VOC profiles. The B-CRSA system consisted of eight quartz crystal microbalance (QCM) sensors that were coated with various metalloporphyrins to promote chemisorption and increase sensitivity to VOCs of interest for lung cancer [69,70]. These were first implemented in the Rome ‘Tor Vertega’ e-nose by D’Amico et al., and are as follows: (1) Ru-meso-TetraPhenylPorphyrin, (2) Rh-[meso-TetraPhenylPorphyrin]-Cl, (3) Mn-[meso-TetraPhenylPorphyrin]-Cl, (4) Co-meso-TetraPhenylPorphyrin, (5) Sn-[meso-TetraPhenylPorphyrin]-Cl2, (6) Co-meso-TetrapNO2 PhenylPorphyrin, (7) Co-meso-Tetra-pOCH3 PhenylPorphyrin, and (8) Mn-OctaMethylCorrole [69,70]. These 8 were chosen based on their good sensitivity (i.e., 100–400 ppb) to aromatic compounds, amines, alcohols, and ketones, and specifically benzene derivatives and alkanes, which have been indicated as possible biomarkers of lung cancer [70]. 

**Figure 2:**
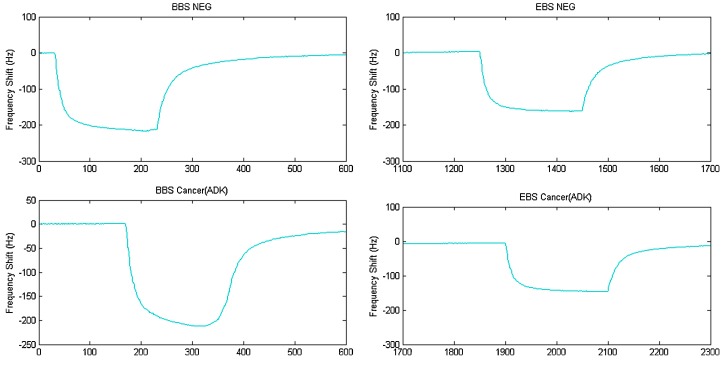
This figure reports the signal registered by Mn-TPP sensor, one of the eight ones composing the sensing array. The first and the second row refer, respectively, to a negative and a cancer individual. The first and the second column refer respectively to the bag breath sampling (BBS) and the endoscopic breath sampling (EBS) sampling techniques. Used with permission from [71].

The sensing mechanism for QCMs is gravimetric: upon analyte(s) binding, the resonant frequencies of the sensors change with respect to the mass of the VOCs present; plotting this frequency shift versus time produces a breathprint for each unique VOC mixture, like the one shown in Figure 2 [71]. In general, the high sensitivity of QCM sensors can be attributed to the high acceleration acting on the deposited film, and the resonant frequency of the crystal is dependent both on the physical properties of the crystal and those of the medium[72]. Sensing properties (i.e., sensitivity and selectivity) of each sensor in the Rome Tor Vertega e-nose array are determined by the central metal and peripheral substituents of the macrocycles in each metalloporphyrin coating. These substituents can be altered easily to produce a variety of metalloporphyrins such as those mentioned above, thereby making it possible fabricate an e-nose device that can be easily altered and optimized to detect target analytes [70]. 

Subjects in the study included 10 subjects with squamous cell carcinoma (SCC), 10 with adenocarcinoma (ADK), and 10 healthy subjects as a control. Two different breath sampling techniques were compared: bag breath sampling (BBS), the standard method, in which subjects breathe into a Tedlar bag through a device designed to catch only deep lung volume (alveolar) air, and endoscopic breath sampling (EBS) near the tumor site via a pump that is connected to an endoscopic probe. A partial least square discriminant analysis (PLS-DA) was then performed for BBS and EBS methods for sample classification and clustering with two different considerations: discriminating between cancer and non-cancer, and between ADK and SCC. Overall, the samples obtained via EBS had superior classification, sensitivity, and selectivity (83%, 87.5%, and 75%, respectively) than that of the BBS method (75%, 81%, 62%, respectively), for the models obtained by separating the data into training and validation sets [71]. While this study presents EBS as an improved alternative to the conventional BBS method, it does not fill the need for a non-invasive diagnosis method for lung cancer. 

QCM sensors in general offer a considerable advantage over conventional gravimetric and other sensing systems in that they are highly sensitive to analytes (with limits of detection on the order of parts per million to parts per billion) and provide a label-free, high-throughput, quantitative approach to analyte detection and vapor analysis; however, they can suffer from instrument drift due to unstable environmental conditions (such as humidity, pressure and temperature) [67,73]. Fortunately, researchers have recently developed more stable and robust QCM systems in order to minimize system inaccuracy while maintaining desired sensitivity including a multi-channel QCM system [73] and a QCM modified with carbon nanotubes [72].

**Figure 3:**
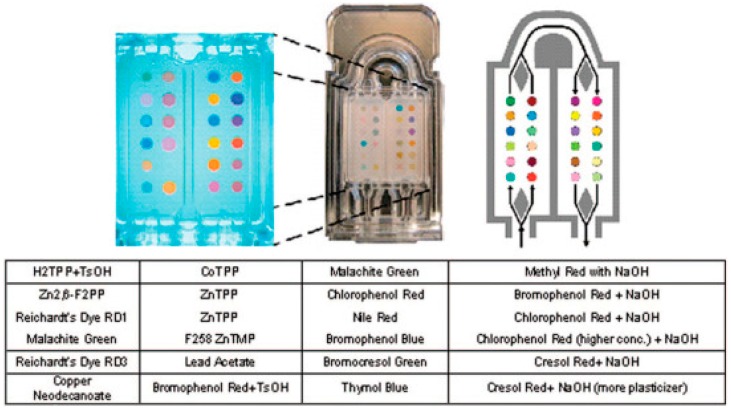
Image of the colorimetric sensor used in this study. Twenty-four chemically reactive colorants are printed on a disposable cartridge. The reactive colorants used in the studied array are listed as they appear on the cartridge. Exhaled breath is drawn across the cartridge in the direction shown. Used with permission from [74].

In 2012, Mazzone et al. developed a portable, inexpensive colorimetric B-CRSA, able to identify lung cancer from healthy controls. The array consists of 24 chemically responsive dyes, printed on a disposable cartridge (see Figure 3). Moreover, in contrast to many of the commercially available e-noses, which can only classify analytes based on a single chemical or physical property, the dyes chosen for the colorimetric array target diverse chemical properties of the analytes, thereby providing a more complete and specific VOC profile. The dyes are divided into three categories: dyes with various metal ions that respond to Lewis basicity, pH indicators that are sensitive to Brønsted acidity/basicity, and dies with large permanent dipoles that respond to various analyte polarities [74].

**Figure 4:**
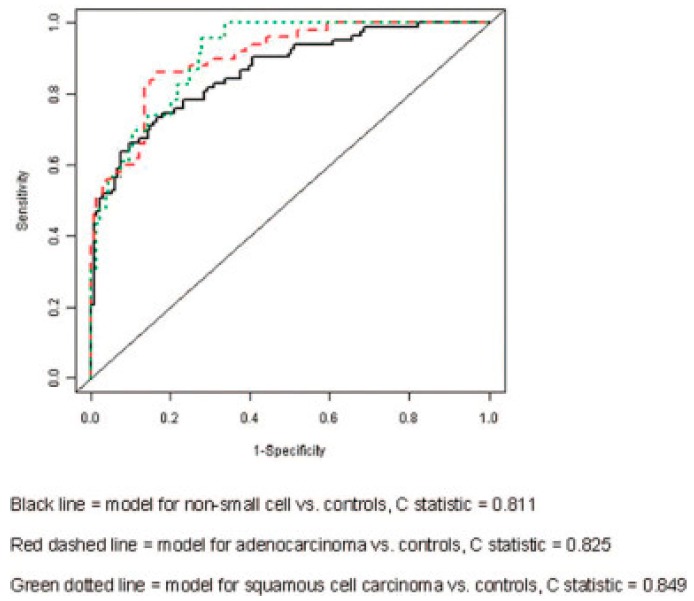
Receiver operating characteristic curves for the most accurate validated models comparing non-small cell carcinoma to controls and for the individual non-small cell carcinoma histologies to controls. Used with permission from [74].

This study also aimed to elucidate breathprints of patients with different types of lung cancer, which had previously not been considered. Biopsy-validated lung cancer subjects were divided into groups according to cancer histology, stage, and survival rate, and their breathprints compared to individuals at risk for developing lung cancer as well as individuals with indeterminate lung nodules. Breath samples were then passed over the array, and images of the array were taken at baseline and at 30 s intervals throughout breath collection. While the array consisted of 24 colorimetric sensors, sensor response was evaluated by converting each image into numerical color change scores for green, red, and blue values, for a total of 72 data points (24 sensors × 3 color change scores). The 72 breathprint data points were then used to construct models to distinguish between groups of interest (see Figure 4), and accuracy of prediction was calculated using C-statistic for that model [74]. The C-statistic is the area under the curve (AUC) of a receiver operating characteristic (ROC) curve, which directly corresponds to the probability of a true positive, accurate prediction, versus a false negative or false positive, with 1.0 being the ideal model [75]. This method was successful at identifying patients with lung cancer versus controls, and showed a higher accuracy when considering the breathprints of specific cancer histologies, rather than global cancer/no cancer response (see Table 1). Overall, this system has great potential to provide a highly accurate, specific, non-invasive, and cost-effective method for improved lung cancer diagnosis.

### 3.2. Colorectal Cancer

Though it is a relatively new direction in metabolomics through B-CRSA analysis, many studies have proposed new, successful methods to non-invasively detect colorectal cancer (CRC). Inspired by the reported success of some canines to identify other types of cancer in humans through sense of smell, a Labrador retriever was put to the test to identify colorectal cancer by smelling the head space of human stool samples [76]. VOCs emitted from feces are a good representation of the types of microbiota present in the colon, making them excellent candidates for CRC biomarkers [36]. The Labrador could identify CRC stool samples from healthy controls with high sensitivity and specificity (91% and 99%, respectively), with an even higher accuracy in watery stool samples (97% sensitivity and 99% specificity) [76]. 

As mentioned in Section 1.1, the gold standard for CRC diagnosis is a colonoscopy. To date, the only alternative clinical method is fecal immunochemical testing (FIT), where fecal samples are screened for known biomarkers of CRC and adenomas (benign tumors formed from glandular tissue); however, this method has very low sensitivity for both CRC and adenomas (66–88% and 27–41%, respectively, depending on the cut-offs used) [49]. CRC exhaled breath VOC profiles have recently been studied through gas-chromatography mass-spectrometry (GC-MS), showing a significant difference between breath profiles of those affected with CRC and healthy controls [77]. GC-MS an ideal method for initial VOC profile analysis to correlate e-nose breathprints with their corresponding VOC profiles for the elucidation of the pathophysiology and the identification of biomarkers; however, it is an impractical approach to diagnostics as it requires specialized and costly equipment, and highly trained personnel. Recent efforts have been directed toward developing e-noses to evaluate CRC in either fecal headspace [49] or urine headspace samples [77] as a more practical approach to olfactory CRC discrimination. 

**Figure 5:**
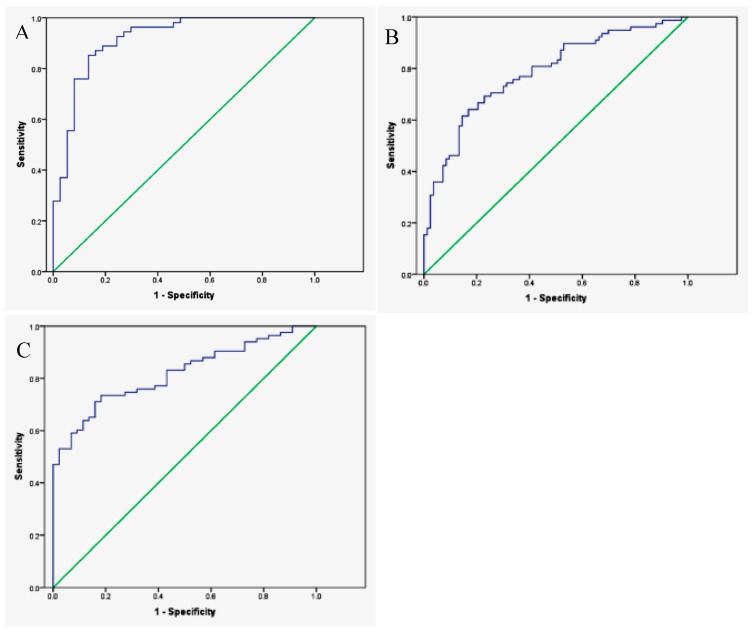
Receiver operating characteristic (ROC)-curves with 95% confidence intervals for diagnosis of (**A**) colorectal cancer (CRC) as compared with advanced adenomas; (**B**) CRC compared with controls; and (**C**) advanced adenomas when compared with controls. Used with permission from [49].

In 2014, de Meij et al. demonstrated that the commercially available Cyranose 320, an e-nose consisting of an array of 32 chemical sensors made of carbon black conducting polymers [32], can successfully distinguish subjects with CRC from those with advanced adenomas and from healthy controls. To perform the study, fecal samples were collected from 40 CRC patients, 60 patients with advanced adenomas, and 57 healthy control subjects. Fecal samples were stored at −20 °C, and *ca*. 2 g of frozen samples were then transferred to a sealed vacutainer and heated to 37 °C for 1 h prior to testing. These containers were then connected to the Cyranose 320 with an airtight closed loop system, including an polyethersulfone syringe water filter and a 3-way stop cock system, to minimize headspace dilution with ambient air, reduce condensation, and to control for flow rate [49]. Cyranose 320 smellprints were then classified using principle component analysis (PCA), a common multivariate data analysis method where the variance of the original dataset is recombined into a set of principle components (PC). Each smellprint is then plotted according to the respective scores of each PC for that smellprint, creating a visual representation of the similarities between certain classes of smellprints [78]. The PCs that represent the largest amount of variance in the data are then used as classifiers, and canonical discriminant analysis (CDA) was performed to calculate the probability of belonging to either of the diagnostic groups for two out of three cases (training set). Resulting algorithms were then validated externally using the remaining cases that were left out of the training set (validation set). This was then repeated with 1000 random distributions of cases for the training and validation set, and the ROC curve (Section 3.1) was plotted for the cross-validated data (see Figure 5). Results were compared to FIT results for the same samples. Overall, the Cyranose 320 could distinguish between each class with good sensitivity and specificity [49].

In 2015, Westenbrink et al. were able to distinguish CRC from healthy controls and those with irritable bowel syndrome (IBS) using an e-nose developed at Warwick University, known as the Warwick Olfaction System (WOLF). The WOLF contains a B-CRSA of 13 sensors that have a variety of different signal transduction mechanisms including 8 amperometric electrochemical sensors, two non-dispersive infrared optical devices, and one photo-ionization detector. The set of sensors was selected such that the device would be sensitive to both gases known to be affected by the body, such as CO_2_, O_2_, and CH_4_, as well as those with a known link to lower gastrointestinal pathology, including NH_3_, SO_2_, and H_2_S. While the B-CRSA is sensitive to these gases, it is also responsive to VOCs with even higher molecular weights. This setup is ideal for an e-nose system, because it maximizes the amount of information for an individual’s volatile smellprint, affording optimal disease classification and differentiation. The WOLF system is housed within a standard-sized PC case, and the results are analyzed and recorded using LabView software. Each sensor is connected to printed circuit boards (PCBs) that convert the raw detection response into an analog voltage level, which is then amplified through a separate PCB via an op-amp circuit. Separate environmental monitors are included to measure flow rate through the machine, as well as temperature and humidity to reduce any effects that could be potentially caused by variance in environment [45]. 

**Figure 6:**
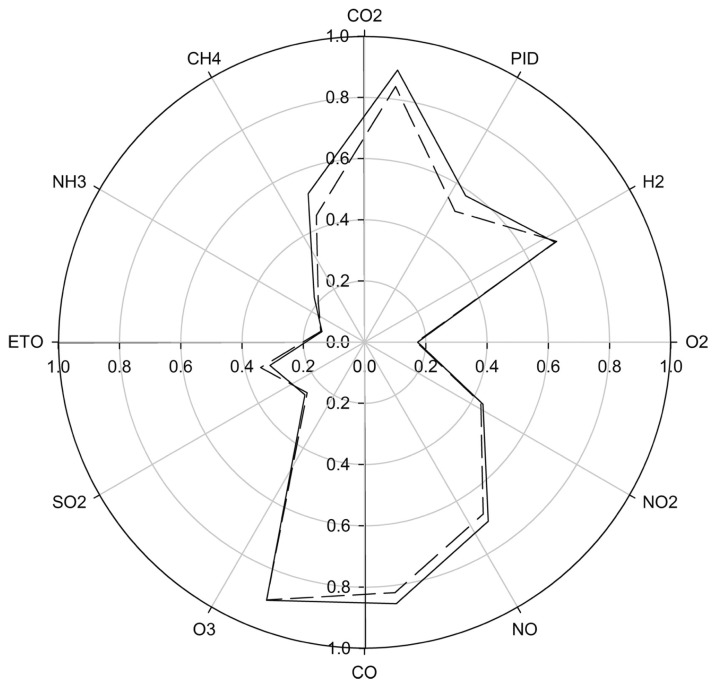
Radial plot of the average normalized response of all 13 Warwick Olfaction System (WOLF) sensors to CRC (full line) and IBS (dashed line) urine samples. Used with permission from [45].

**Figure 7:**
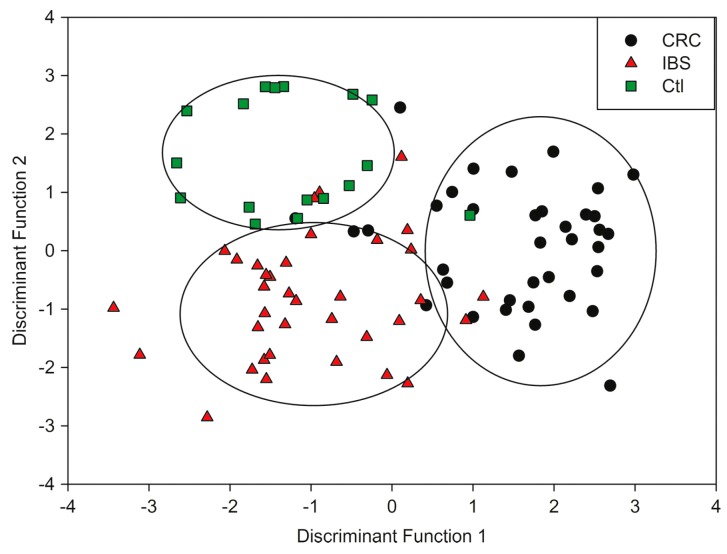
LDA classification separating all three sample groups of CRC, Irritable Bowel Syndrome (IBS) and healthy controls (Ctl). Used with permission from [45].

The study contained three sample groups, 39 suffering from CRC, 35 suffering from IBS, and 18 healthy controls. Urine samples were collected from subjects as standard spot urine early in the morning, and “dipstick” tests ruled out the presence of infection, diabetes, or renal disease. A flow of clean dry air was split into two channels, and one directed to the headspace of a 5 mL sample aliquot, heated to 40 °C for 5 min. A check-valve was incorporated into the sample channel before joining again with the other “make-up” channel, to ensure stable pressure and flow rate and reduce consequential effects on sensor response. Mixed air from both channels was then injected into the WOLF system for 5 min to ensure that the sensors reached equilibrium, and all of the samples were analyzed in triplicate to evaluate reproducibility. The smellprints of collective sensor responses were constructed by extracting three features from the raw data: (1) baseline-corrected voltage changes averaged over three points; (2) response integrals from start of response to maximum response; and (3) times for sensor responses to return from a maximum to 50% of that value. The smellprints were classified for each sample group via Linear Discriminate Analysis (LDA), which is a technique to discriminate classes, i.e., “use the information in a learning set of labeled observations to construct a classifier (or classification rule) that will separate the predefined classes as much as possible”, and then classify unknown, new samples using “the classifier to predict the class of that observation” [79]. Figure 6 shows the normalized changes in voltage for each of the 13 sensors for CRC and IBD subjects, and LDA plots are shown in Figure 7. As the figures suggest, the WOLF system successfully differentiated these two groups from each other as well as healthy controls, with a sensitivity of 78% and specificity of 79% [45]. While these numbers are an improvement from previous non-invasive diagnostic techniques for CRC, there is still room for sensor optimization to increase accuracy of diagnosis. 

### 3.3. Head and Neck Cancer

Head and neck cancer (HNC) comprises *ca*. 3% of cancer cases in the U.S., with a projected 63,030 new cases developing in 2017 [5,80]. The most common type of HNC is head and neck squamous cell carcinoma (HNSCC), which comprises 90% of cases and is usually treated with both surgery and radiotherapy [81,82]. While technological advances have improved both the treatment and diagnosis methods in the last twenty years, little improvement has been made in the overall survival rate [80]. Furthermore, many HNC cases occur in developing countries, with limited access to specialized equipment and healthcare [83]. Current HNC diagnosis is performed via panendoscopy, which is a highly invasive procedure including “rigid tracheobronchoscopy, rigid esophagoscopy, direct laryngoscopy, hypopharyngoscopy, and inspection and palpation of the oral cavity and the oropharynx—with a subsequent biopsy for histopathological examination under general anaesthesia” [81]. Thus, there remains a need for a non-invasive HNC diagnosis method that would afford early detection and intervention while maximizing affordability, ease-of-use, and throughput.

HNC is a great candidate for diagnosis via e-nose exhaled breath analysis because tumors are in proximity to the oral cavity and airways, and the VOCs released from compromised metabolic processes of diseased tumor cells mix with breath upon exiting the oral cavity. One study performed between 2010 and 2013 examined 36 patients with histopathologically-confirmed HNSCC, including cancer of the oropharynx, hypopharynx, and supraglottic larynx, with a commercially available e-nose known as the DiagNose [81]. The DiagNose system B-CRSA consists of 12 metal-oxide sensors of four types (CH_4_, CO, NOx, Pt) in triplicate. This system monitors changes of resistance for each of the sensors upon both adsorption and desorption of the VOC mixture. Samples of air are pumped in from a sample bag at one inlet, and a second inlet pumps in filtered ambient air for baseline measurement. Air composition is measured every 20 s via a 32-step sinusoidal modulation of the sensor surface temperature (correlated to electrical resistance), thereby producing a 32-dimensional vector every 20 s for each of the 12 sensors. In total, the measurement runs for 10 min: the first 5 min evaluating VOC adsorption and the second 5 min evaluating VOC desorption characteristics.

HNSCC breath samples (100% smokers) were compared to breath samples of subjects without cancer, but who were active smokers. Sensor data for each sample was downloaded and processed as follows: potential pollution was accounted for by deleting the first and last sections of collection, and data was then normalized to minimize internal sensor differences by Equation 1, where *Xnorm* is the normalized data point, *xt* is the raw measurement at time point *t*, and *T* refers to the observed temperatures.

(1) $$X_{norm}=\frac{x_{t}}{\Delta T-T_{min}}$$

**Figure 8:**
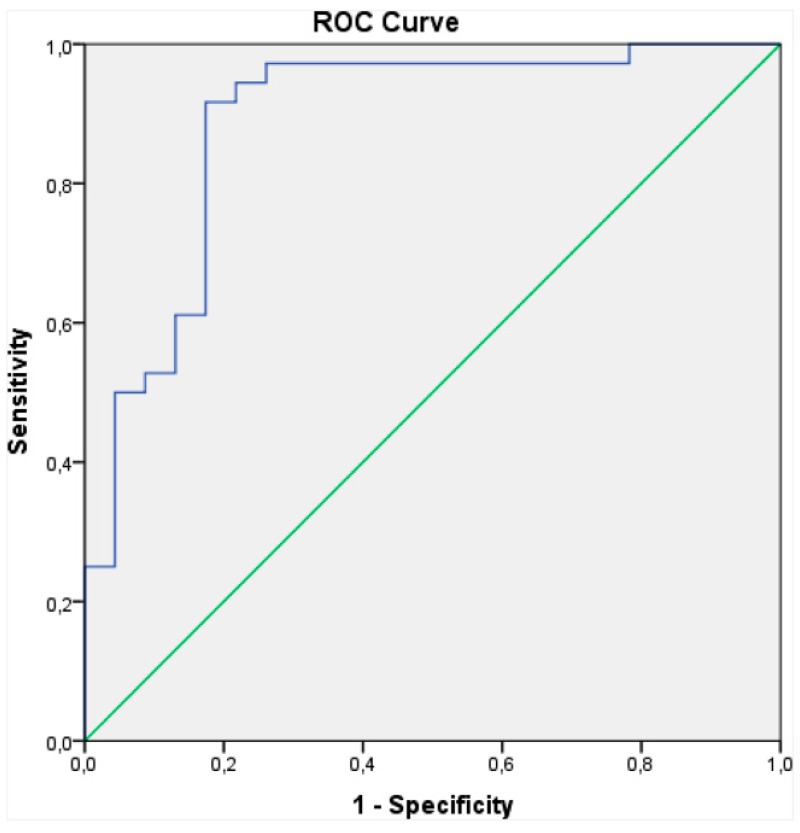
Data points from sensor measurements were used to construct a ROC-curve. Logistic regression showed a significant difference (*p* < 0.05) in VOC resistance patterns between patients diagnosed with head and neck squamous cell carcinoma (HNSCC) and the control group, with a sensitivity of 90% and a specificity of 80%. HNSCC head and neck squamous cell carcinoma; ROC receiver operating characteristic; VOC volatile organic compounds. Commas in axis numbers represent decimal places. Used with permission from [81].

The AUC (Section 3.1) was also calculated for each sensor, and the mean of each of the four sensor types was taken. A logistic regression was then performed on the resulting 128 values (32 data points × 4 sensor types) correlating sensor readouts to diseased/healthy samples, and an ROC curve (Section 3.1) was plotted (Figure 8). Logistic regression analysis showed successful differentiation of HNSCC for each sensor type, with an overall sensitivity of 90% and specificity of 80% [81]. With such good results, the DiagNose system certainly offers great potential for an alternative diagnosis method that is non-invasive, fast, and affordable, though a larger study is needed to confirm results for HNSCC and other types of HNC before it can be implemented. 

### 3.4. Prostate Cancer

Prostate cancer (PC) is the second most common type of cancer among males, and though tumor progression is usually slow, it is still associated with a high mortality rate [84]. Additionally, PC is notoriously difficult to diagnose due to heterogeneity, which also makes it difficult to estimate an accurate prognosis. Currently, screening and diagnostic methods for PC include digital rectal exam (DRE) and plasma prostate-specific antigen (PSA), although these methods have limited sensitivity. The only definitive diagnosis existing today is histological examination of transrectal ultrasound guided biopsy, which is costly, very uncomfortable for the patient, and carries a risk of infectious complications [33]. There have been many studies on prostate cancer urine biomarkers to improve diagnosis accuracy and develop alternative non-invasive methods for detecting the disease; however, many of these methods still require expensive and specialized equipment or lengthy assays [35]. The implementation of an e-nose (for urine headspace) or e-tongue (for biomarkers in body fluids) would considerably cut down on the cost and time of analysis. By correlating smellprint or tasteprint of the devices with known biomarker profiles of patient urine samples determined in preliminary studies, future studies could be conducted to quickly identify biomarkers for personalized treatment without the need for in-depth analysis with specialized equipment. 

In 2014, Roine et al. performed a study to detect PC with a commercially available e-nose via urine headspace samples. The study included 50 patients with histologically-confirmed PC and 24 control samples of 15 subjects with benign protastic hyperplasia (BPH) [33]. The e-nose was a ChemPro^®^ system, developed in Finland, consisting of 8 electrode strips, each producing a two-channel output and a MOS cell [85], providing 18 data points for evaluation of each sample. The measurement chamber was built in-house, and made of a polystyrene cell culture plate, with a parafilm-secured cover in which 3 holes are drilled. Two of the holes serve to replace air after drawing out headspace air, and the third hole contains a 16 G cannula with the injection port replaced by a Teflon tube, which acts as the inlet for the ChemPro e-nose. Each sample (5 mL) was defrosted and deposited in the culture plate, creating a wide, thin layer of fluid; this increases sample exposure to air and therefore maximizes headspace VOC concentration [33]. 

**Figure 9:**
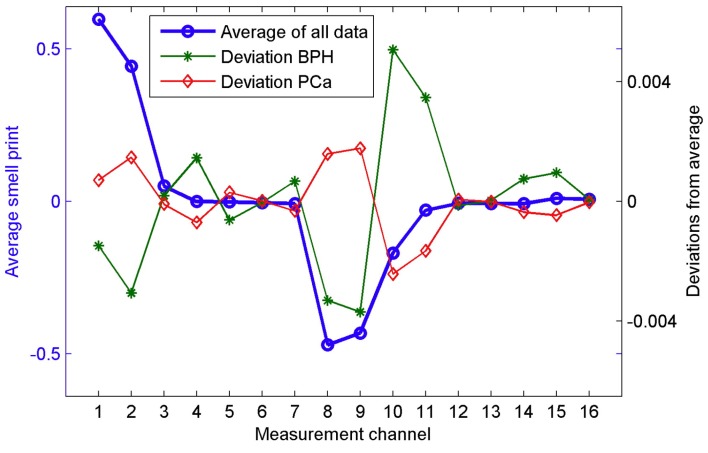
Total average of all data represents typical smell print of urine. Characteristics of average smell prints of benign protastic hyperplasia (BPH) and principal component analyses (PCA) groups are depicted by deviation from total average. Used with permission from [33].

**Figure 10:**
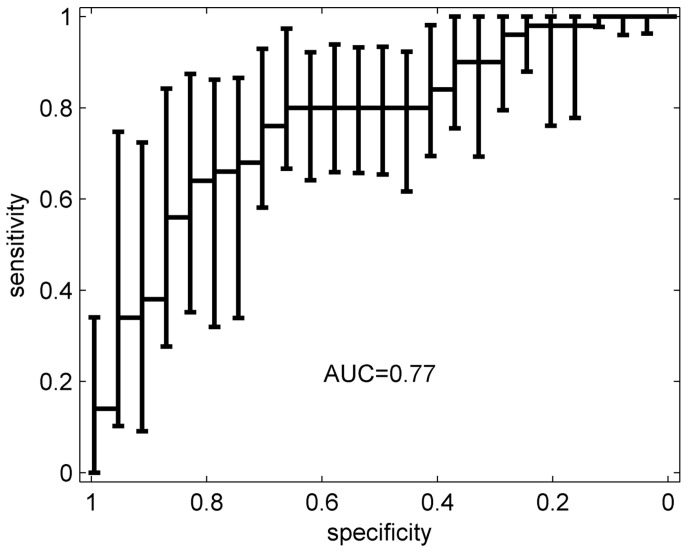
Total average of all data represents typical smell print of urine. Characteristics of average smell prints of BPH and PCA groups are depicted by deviation from total average. Used with permission from [33].

During the 15-min sampling period, maximum absolute values of sensor resistance change were extracted for further analysis. All but two of the channels had responses, resulting in 16 data points for each sample. Resulting average smellprints for each group are shown in Figure 9. Sample data was then scaled by dividing each element (16 channels) with the L2 norm (magnitude) of the entire sample vector, thereby making the L2 norm of each sample vector 1. LDA (Section 3.2) was then used to assign samples to one of two classes: PC and BPH, and standard statistical methods were employed to reduce over-fitting (leave-one-out cross validation) and to correct for bias. Additionally, a multilinear regression model was constructed to correlate prostate volume with BPH cases and tumor size with PC cases to predict prostate size and tumor size, respectively, using e-nose data. Finally, an ROC curve was constructed and the AUC value calculated (Section 3.1) to evaluate e-nose sensitivity and specificity, as shown in Figure 10 [33].

The ChemPro could identify prostate cancer with 78% sensitivity and 67% specificity after cross-validation was performed to reduce overfitting and to more accurately model an actual diagnostic implementation. While no significant model could be developed to predict tumor size, channel 9 on the e-nose showed statistically significant correlation (*p* = 0.02, correlation coefficient of 0.34) with prostate size for BPH samples [33]. This proof of principle study shows the potential for e-nose diagnosis of prostate cancer through urine headspace, though there is still room for improvements in both sensitivity and specificity. Further studies, perhaps with a wider variety of sensors, are needed to optimize e-nose diagnosis of prostate cancer.

## 4. Airway Diseases

Lung diseases were some of the first to be evaluated via exhaled breathprint profiling with e-nose devices [2,32]. Lung diseases fall into two distinct categories: obstructive and restrictive. Obstructive diseases are any lung diseases that cause blockage in the airways, including lung damage and narrowing of airways due to blockage, inflammation or excess mucous. Restrictive diseases are those that affect the ability of the lung to expand to full capacity, such as stiffening of lung tissue or reduction of chest cavity volume due to physical conditions such as scoliosis or obesity. While past studies have successfully profiled and classified a variety of lung diseases, there remains a need for further discrimination abilities (specificity) using e-noses to determine disease stage and comorbidity.

### 4.1. Chronic Obstructive Pulmonary Disease (COPD) 

COPD specifically encompasses a group of “progressive, debilitating lung conditions, including emphysema and chronic bronchitis, characterized by difficulty breathing, lung airflow limitations, cough, and other symptoms” [86]. The current gold standard for diagnosis is via spirometry, a measurement of flow volume loop upon a full breath cycle after administration of bronchodilators. From the flow volume loop, the ratio of forced expiratory volume in the first second (FEV1) to forced vital capacity (FVC, the maximum total amount of air that can be forcefully expired after maximum inhalation) is measured, and an FEV1/FVC ratio <0.7 confirms the presence of persistent airflow limitation, thus confirming COPD diagnosis [87]. While this method is minimally invasive and it is a good measure for determining COPD severity, it cannot provide information about pathogenesis of disease nor presence of infection. Distal airway infection diagnosis must be performed by quantitative culture of protected specimen brush (PSB)—a procedure that is limited by both a lengthy processing time and invasive sampling procedure [88]. Treatment methods for COPD largely vary depending on patient response (trial and error), specific disease type, and severity, and there is currently no treatment that has been effective at reducing the long term decline in lung function [87]. Thus, recent efforts have been focused on identifying the specific underlying pathophysiological processes in individual cases [88], evaluating the breathprints of COPD patients with comorbid diseases such as obstructive sleep apnea (OSA) [89], and within-day and between-day variations in breathprints of COPD patients [90].

To address the issue of the invasive procedure that is required to confirm distal airway infections, Sibila et al. have proposed the alternative, non-invasive method of airway infection diagnosis via VOC breathprint analysis of exhaled breath with the Cyranose 320 (Section 3.2). In this study, breathprints were obtained from 37 clinically stable COPD patients (10 with confirmed airway bacterial colonization) and 13 healthy controls. Exhaled breath samples were obtained prior to bronchoscopic procedures. Individuals breathed into a 10 L Tedlar bag after 3 min of tidal breathing through an inspiratory filter, and expiratory silica reservoir exposed to dry air. The Cyranose 320 was then connected to the sample bag and sensors were exposed to breath samples for 5 min [88]. 

**Figure 11:**
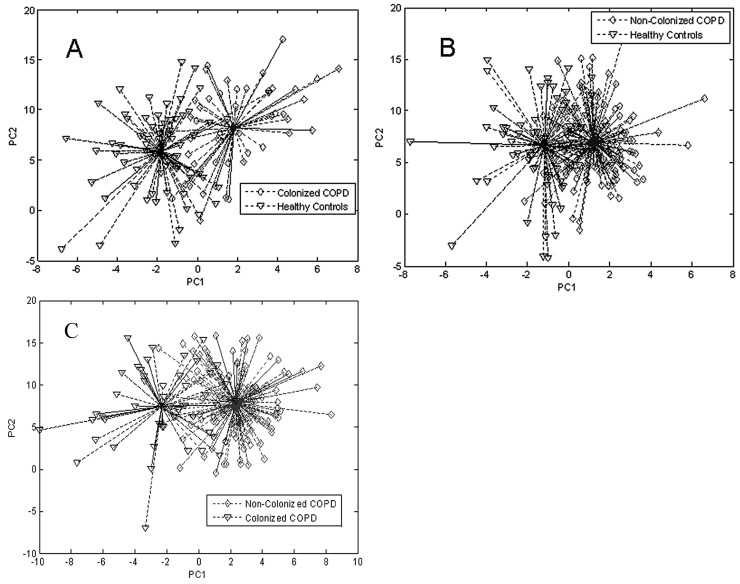
Two-dimensional principal component analyses (PCA) plot showing the discrimination of breath-prints in (**A**) colonized chronic obstructive pulmonary disease (COPD) patients and healthy controls; (**B**) Non-colonized COPD patients and healthy controls; and (**C**) colonized COPD patients and non-colonized COPD patients. Used with permission from [88].

**Figure 12:**
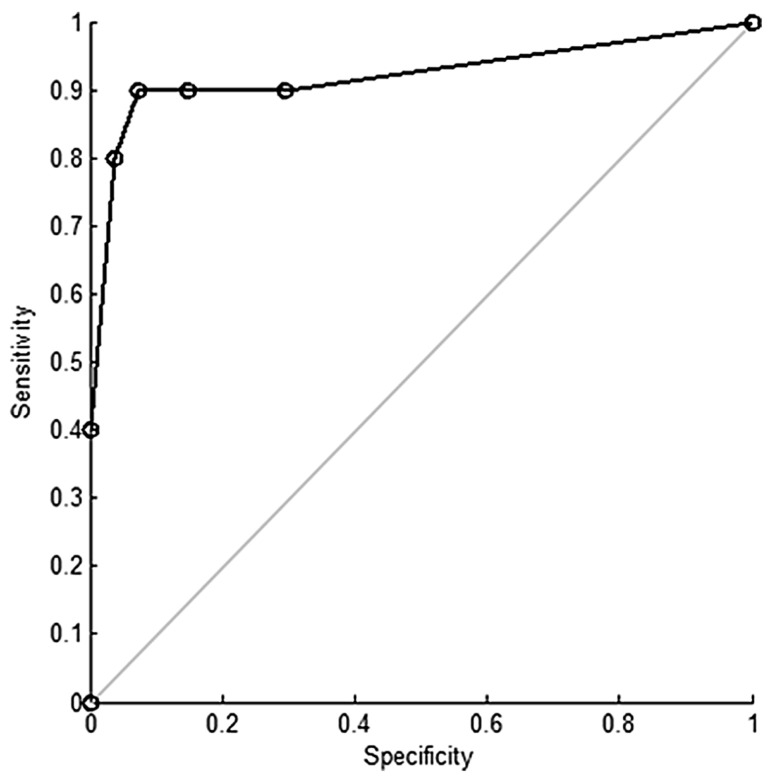
Receiver operating characteristic (ROC) curve of the model predicting the presence of bacterial airway colonization in COPD patients. Used with permission from [88].

Raw data from the Cyranose 320 sensors was reduced via PCA (Section 3.2) and then processed using a pattern recognition application in Matlab. PCA plots are shown in Figure 11. The Matlab software employs LDA (Section 3.2) for patient classification, and verifies classification model accuracy by leave-one-out cross validation. The ROC curve (Section 3.1) was then constructed to evaluate sensitivity and specificity of the device for each sample class, and is plotted in Figure 12. The Cyranose 320 could successfully distinguish between all 3 sample classes—non-colonized COPD, colonized COPD, and healthy controls—with a high degree of sensitivity and specificity as shown in Table 2. It is important to note that the degree of specificity using the Cyranose 320 is high between colonized and non-colonized COPD patients (0.96), indicating that this method shows much promise as an alternative, non-invasive way of detecting bacterial colonization for diagnosing distal airway infections [88].

### 4.2. Obstructive Sleep Apnea (OSA)

OSA is characterized by episodes of partial and complete airway obstruction, resulting in multiple apneas (temporary cessation of breathing) and hypopneas (abnormally slow or shallow breathing). It is becoming a widespread concern worldwide, with an increased number of cases diagnosed annually in both men and women [7], and is associated with an increased risk of cardiovascular diseases and metabolic disorders [41]. Currently, the gold standard for OSA diagnosis is performing multichannel polysomnography (PSG), which is an in-patient sleep study that requires the monitoring of several parameters, including “snoring, apneas, nocturnal choking or gasping, restlessness, and excessive daytime sleepiness” and an extensive review of sleep history [91]. This technique is altogether uncomfortable for the patient, lengthy, and costly, with limited availability. Some alternative methods have been proposed to cut down on labor and cost, such as the Epworth Sleepiness Scale, neck circumference, and comprehensive clinical score; however, these lack both specificity and sensitivity with a great degree of overlap between those with OSA and healthy controls [92]. 

Some recent studies have focused on the elucidation of OSA biomarkers that are associated with oxidative stress and both systemic and airway inflammation [92]. Both oxidative stress and inflammation have been known to produce VOCs in exhaled breath [32,68]. Based on this principle, Greulich et al. performed a proof-of-principle study to identify OSA by exhaled VOC profiling, with an e-nose as an alternative method for OSA diagnosis and as a way of monitoring the effectiveness of treatment. The study consisted of 20 healthy volunteers and 40 with previously diagnosed OSA. Those with OSA had not yet received therapy via continuous positive airway pressure (CPAP), which is prescribed to keep airways open during sleep to normalize breathing, reduce apneas and hypopneas, and reduce airway inflammation.

**Figure 13:**
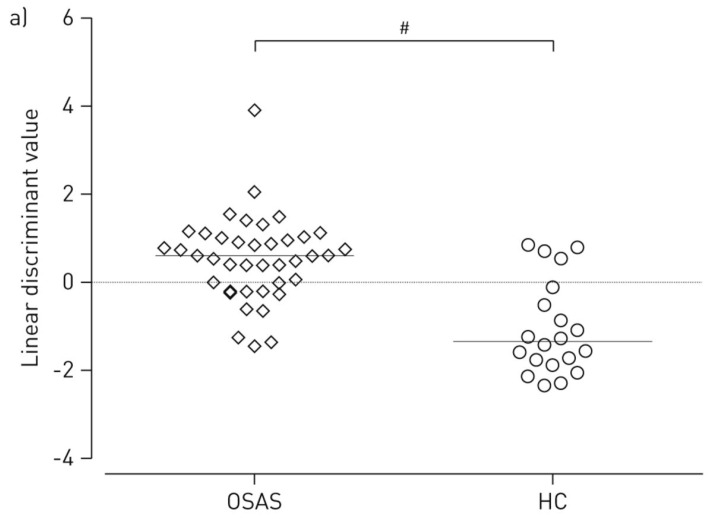

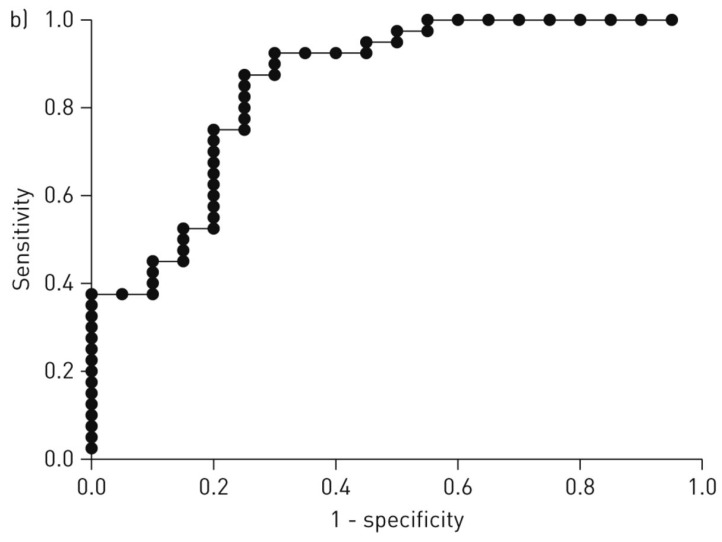
(**a**) Linear discriminant analysis of sleep apnoea patients and healthy controls differ statistically significantly. (**b**) The area under the receiver operating characteristic curve equals 0.85, resulting in a sensitivity of 0.93 and a specificity of 0.70. OSAS: obstructive sleep apnoea syndrome; HC: healthy control. #: *p* < 0.0001, Mann–Whitney U-test. Used with permission from [92].

Exhaled breath samples were evaluated in triplicate with the Cyranose 320 e-nose. The three data sets obtained were then averaged, and PCA performed (Section 3.1) to capture the largest variance between sets before modelling with LDA (Section 3.2). LDA models were verified via leave-one-out cross validation, and an ROC curve was constructed (Section 3.1) to calculate specificity and sensitivity for each sample class. To evaluate inflammation levels before and after CPAP therapy, pH, conductivity of exhaled breath condensate (EBC), and the inflammatory biomarkers matrix metalloproteases (MMPs), tissue inhibitor of metalloproteases (TIMPs), and α1-antitripsin (α1-AT) were measured in pharyngeal washing fluid. LDA for e-nose data revealed a statistically significant difference between healthy controls and patients with OSA, as shown in Figure 13, and a sensitivity of 0.93 and specificity of 0.70 was calculated with the AUC for the ROC curve [92]. 

**Figure 14:**
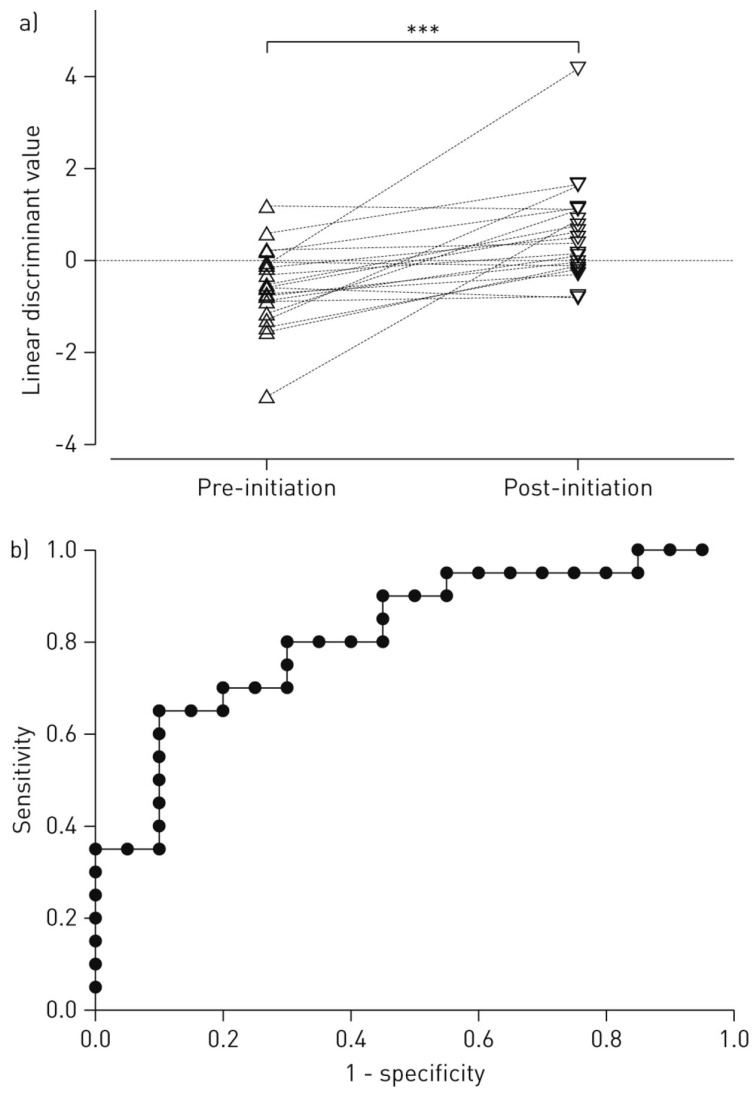
(**a**) The (paired) values of the linear discriminant analysis of sleep apnoea patients before (pre-initiation) and three months after (post-initiation) initiation of continuous positive airway pressure therapy differ statistical significantly. (**b**) The area under the receiver operating characteristic curve equals 0.82, resulting in a sensitivity of 0.80 and a specificity of 0.65. ***: *p* < 0.001, Wilcoxon signed-rank test. Used with permission from [92].

While most of the inflammatory biomarkers (pH, EBC conductivity, MMP-9, and TIMP-1) did not reach statistical significance between healthy controls and OSA patients, there was a statistically significant difference of α1-AT levels in pharyngeal washing fluid. Compared with LDA predictive models built with e-nose VOC data, the predictive models built using data for inflammatory markers were not as accurate; however, 100% accurate prediction was possible by combining e-nose LDA with inflammatory marker data. Interestingly, e-nose LDA models for the first 20 OSA patients showed a significant difference between pre-therapy and post-therapy VOC profiles evaluated after three months of CPAP treatment, as shown in Figure 14, with a sensitivity of 0.80 and specificity of 0.65. Regarding inflammatory markers, EBC conductivity was significantly different between pre- and post-therapy, and post-therapy levels of α1-AT were significantly lower [92]. 

Results from this study show that diagnosis and monitoring of OSA with e-nose technology shows much promise as an alternative to PSG. While the current AUC value of 0.85 is not sufficient for a definitive, comprehensive diagnosis, the Cyranose 320 could still be used as a valuable screening tool for low-risk populations, with a negative predictive value of 99.6% [92]. In higher risk populations, such as those with obesity, the results may not be as accurate [41]; however, the Cyranose 320 could still be used as a tool for determining the need for PSG testing. As discussed previously, the Cyranose 320 system contains a B-CRSA with sensors of one type of signal transduction mechanism. Thus, further studies should be conducted with different types of e-nose sensors to improve sensitivity and specificity for a more accurate diagnosis. 

### 4.3. Pulmonary Sarcoidosis (PS) 

Falling within the class of restrictive lung diseases, PS is characterized by the formation of granulomas within the lung, for which the cause is unknown. These granulomas greatly limit lung expansion and airflow, and they have been known to form in both young and middle-aged adults worldwide [93]. Not only is the cause of the granulomas unknown, but also the disease progression and clinical presentation vary widely among patients, making diagnosis and prognosis difficult. Indeed, most cases require invasive methods such as bronchoscopy for definitive diagnosis [94]. 

**Figure 15:**
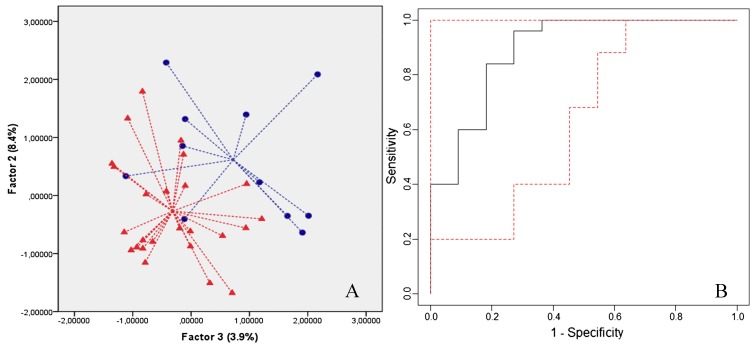
(**A**) Two-dimensional principal component analysis with two composite factors showing the discrimination of breathprints between patients with untreated sarcoidosis (blue circles) and controls (red triangles) and (**B**) ROC-curve with 95% confidence interval for diagnosis of untreated sarcoidosis compared to controls. AUC was 0.825. Comma between numbers in (A) represent decimal places. Used with permission from [94].

In 2013, a study was conducted by Dragonieri et al. to evaluate e-nose ability to distinguish between subjects with treated PS, untreated PS, and healthy controls. The Cyranose 320 was again selected as the commercially available e-nose of choice, and data analysis, including PCA, LDA predictive modelling, and ROC curve analysis was performed, as described in previous sections. The study consisted of 11 patients with untreated PS (recently diagnosed), 20 with treated PS (in various stages), and 25 healthy controls, and duplicate samples of each person were evaluated to ensure the reproducibility of results. Overall, the Cyranose 320 could distinguish between VOC breathprints of patients with untreated PS and healthy controls, with an LDA cross validation accuracy (CVA) of 83.3%. Breathprints of treated versus untreated PS were barely distinguishable with a CVA of 74.2%, and breathprints of treated PS could not accurately be distinguished from healthy controls. The PCA plot of untreated PS vs. healthy controls, and the corresponding ROC curve is shown in Figure 15 [94]. The success of the Cyranose 320 to distinguish untreated PS from healthy controls shows its potential for a quick, non-invasive PS diagnostic method. Furthermore, the inability of the Cyranose 320 to distinguish treated patients from healthy controls suggests that it could be used to monitor the effectiveness of treatments: if VOCs are largely produced by oxidative stress and inflammation that causes tissue damage and a lack of function, then the reduced levels of these VOCs in the treated PS patients’ breath samples implies that the therapy is successful to a certain degree.

## 5. Neurodegenerative Diseases and Mental Health

### 5.1. Alzheimer’s Disease (AD) and Parkinson’s Disease (PD)

AD and PD are two of the most common neurodegenerative diseases today. As the population of people ages 65 and over increases, AD and PD prevalence will also increase. In 2016, an estimated 5.4 million Americans were diagnosed with AD, 200,000 of which had early onset AD and were under the age of 65. For those over the age of 65, AD prevalence was 1 in 9 people (11%), and 1 in 3 for those over the age of 85 [95]. PD prevalence also rises with age, from 41 individuals per 100,000 ages 40–49 to 1903 individuals per 100,000 over the age of 80 [96]. While both diseases are neurodegenerative in nature, AD is largely characterized by a progressive cognitive and behavioral deterioration, and PD mainly affects motor skills, including resting tremor, bradykinesia, rigidity, and postural instability [37]. Currently, there is no definitive, all-inclusive procedure for AD or PD diagnosis, and methods rely on collaborative efforts from a physician and neurologist to evaluate family medical history, changes in thinking and behavior, a series of cognitive and physical tests, blood tests, and brain imaging [95,97]. Recent efforts have been directed toward AD and PD metabolomics to elucidate definitive biomarkers for each disease [97]; however, these are largely in serum, blood, and cerebrospinal fluid, all of which require invasive procedures to obtain. Moreover, recent findings suggest that neurodegeneration begins several years before the onset of the symptoms on which clinical diagnoses depend [37]. Therefore, there remains a need for a non-invasive, high throughput, early detection method for diagnosing AD and PD definitively. 

In 2013, Tisch et al. performed a proof-of-principle study with a nanomaterial-based B-CRSA to detect AD and PD from each other and healthy controls [37]. These sensors were previously successful at diagnosing multiple sclerosis [98], another debilitating neurodegenerative disease. The study included a total of 57 non-smoking volunteers that were aged 37–82 years, in which there were 15 AD patients, 30 PD patients, and 12 healthy controls. Breath samples were collected from each person after breathing through a filter for 5 min to eliminate ambient inhaled VOCs. Because alveolar air contains the VOCs of interest, dead space air (the first half of the volume of expired air) was collected in a separate bag and discarded, while alveolar air was filled into a separate Mylar bag for sampling. This is a one step process that does not require the volunteer to change the bags while exhaling. Breath samples were analyzed on the same day of collection both by GC-MS to determine specific VOCs present and by the B-CRSA to obtain the VOC breath print for each person. 

The B-CRSA consisted of an array of 20 organically-functionalized nanomaterial based sensors in a stainless-steel exposure chamber, which exhibited a rapid and reversible change in resistance upon VOC binding and absorption. Overall, there were six different types of sensors with different organic functionalities (described in Table 3) and base materials including random networks of single-walled carbon nanotubes and gold nanoparticles. The organic functionalities “provided broadly cross-reactive absorption sites for breath VOCs”, and sensors have rapid response time (within 5 s of exposure) and high sensitivity, picking up concentrations on the order of parts per billion (ppb). Four parameters were measured for each sensor: normalized resistance change at the middle (1) and end (2) of exposure time, and the area under the response curve at the beginning (3) and end (4) of the signal [37]. 

**Figure 16:**
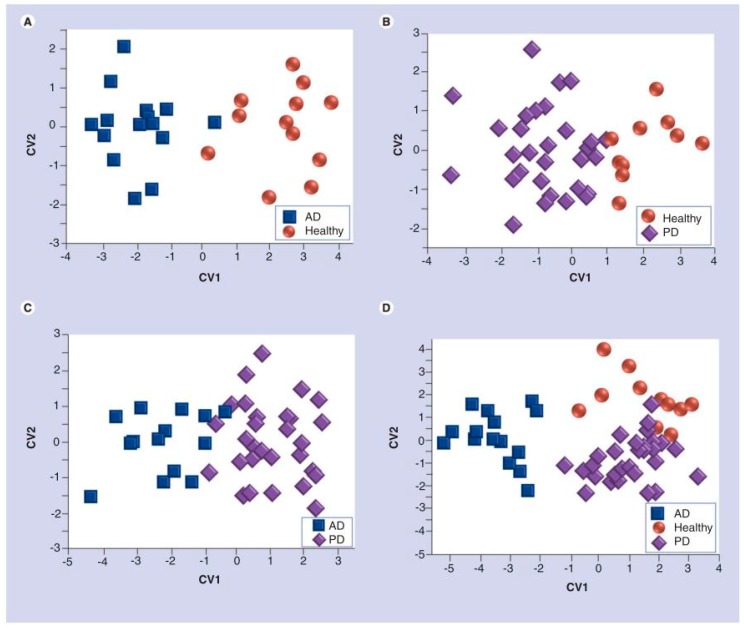
Discriminant factor analysis plots. CV1 and CV2 were calculated from the sensing responses to (**A**) the Alzheimer’s Disease (AD) patient population and healthy controls; (**B**) the Parkinson’s Disease (PD) patient population and healthy controls; (**C**) the AD and PD patients; and (**D**) the AD and PD patients and healthy controls. Each patient is represented by one point in the plot. AD: Alzheimer’s disease; CV1: First canonical variable; CV2: Second canonical variable; PD: Parkinson’s disease. Used with permission from [37].

After exposure to breath samples, discriminate factor analysis (DFA) was performed using the sensor data to select the most suitable set of sensing features to represent a collective array response and construct breathprints for each sample class that could be easily distinguished from other classes (maximum variance of sensor responses for each sample class). DFA is like LDA and PCA in that it is a linear, supervised pattern recognition method that aims to reduce the dimensions of the data into canonical variables (CVs) that maximize the variance between classes and minimize the variance within classes [99]. For discrimination between classes, the first and second CVs are usually sufficient for clustering the data correctly. In this study specifically, DFA was used to select the three most suitable sensors that represented the optimal separation between classes of breath samples. This was done to limit over-fitting of the model, and leave-one-out cross validation was performed to ensure classification accuracy. Three DFA models were built in this way: (1) to distinguish AD from healthy controls; (2) to distinguish PD from healthy controls; and, (3) to distinguish AD from PD. The first model was accurate in identifying AD samples, with a sensitivity, specificity, and accuracy of 93%, 75%, and 83%, respectively. The second model had similar success in identifying PD patients, with a sensitivity, specificity, and accuracy of 70%, 100%, and 79%, respectively. Finally, the third model also demonstrated the success of the B-CRSA in separating AD from PD patients, with a sensitivity, specificity, and accuracy of 80%, 87%, and 84%, respectively. DFA plots of CV1 vs. CV2 are shown in Figure 16 [37]. 

**Figure 17:**
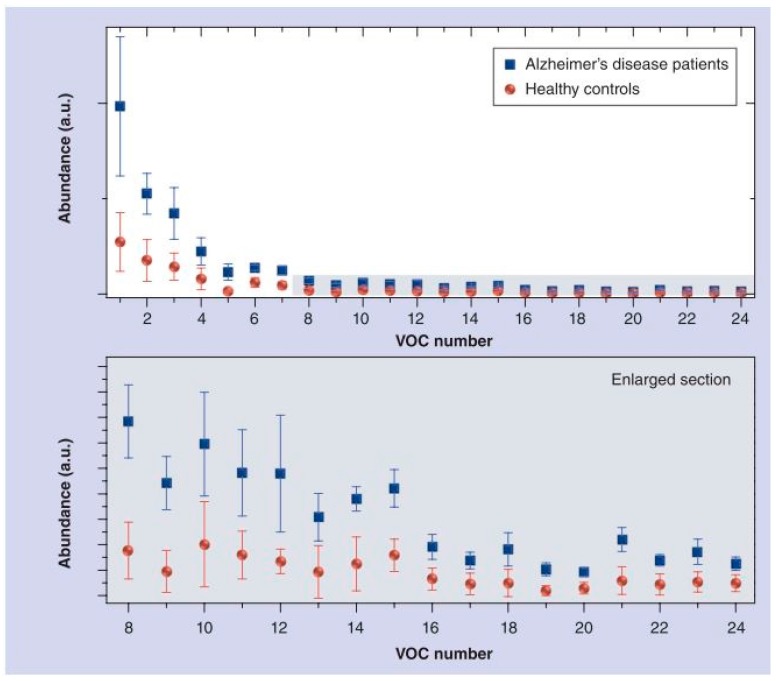
Gas chromatography-mass spectrometry analysis of the breath of Alzheimer’s disease patients. Abundance of 24 VOCs that were found in the breath of >80% of both the Alzheimer’s disease patients and the healthy controls. The symbols represent the average abundance and the error bars mark the borders of the 95% CIs. VOC: Volatile organic compound. Used with permission from [37].

This study greatly demonstrates the success of a B-CRSA in identifying AD and PD from healthy controls and each other, suggesting that exhaled breath could be used in the future as a rapid, non-invasive diagnosis method for these diseases. Moreover, GC-MS results revealed 24 distinct VOCs for AD and 7 VOCs for PD with significantly different concentrations than the control breath samples, as shown in Figure 17 [37]. Using this data, a library of corresponding breathprints to VOC profiles could be constructed for reference in future e-nose diagnostics, thereby eliminating the need for specialized and costly GC-MS analysis every time.

### 5.2. Future Direction: Chronic Stress and Anxiety

Collectively, anxiety disorders are the most common mental health problem in the United States, affecting approximately 18% of adults in the general population per year, and 29% of adults at some point during their lifetime [100]. Although anxiety itself is an adaptive and universal human reaction to stressful situations, in excess it is both distressing and impairing. The descriptive nosology embodied in the Diagnostic and Statistical Manual (DSM) has provided the field with a common language for establishing reliable anxiety diagnoses [101]; however, there is widespread consensus that the DSM’s categorical syndromes have significant limitations. It is likely that underlying biological processes linked to the persistence and severity of the often overlapping core components of anxiety syndromes are linked to cross cutting specific features rather than diagnoses. While the next two studies presented here do not employ B-CRSA technology, they demonstrate that emitted metabolites can be correlated to emotional and stress response, and provide a rationale for future studies to be performed with B-CRSA technology to evaluate emotional and stress response of VOCs, while reducing cost of analysis and the need for specialized equipment.

Recently, many advances have been made in diagnostic identification methodology for a variety of diseases as technological advances in research have enabled the identification of biomarkers as targets for detection, diagnosis, and therapy. Mental health research has taken a similar approach; new findings in mental illness research have shown that current diagnostic methods are insufficient because many markers of mental illness, such as risk genes and metabolites, are associated with more than one mental illness [102]. Unlike cancer and cardiovascular diseases, biological findings for mental illness suggest that there is not a one-to-one mapping of a specific biomarker to one illness. Thus, recent efforts have been directed toward targeting a specific category of mental pathophysiology, such as risk genes, cell morphology, metabolites, and others, whereby a disease profile can be identified by recording multiple data points within a category. While there are many parallels in biological data associated with mental illness, the unique combinations of biomarkers for each disease enable classification. By correlating the obtained biological profile of a specific patient with clinical observations and reported symptoms, a more specific and accurate diagnosis can be achieved.

Salivary inflammatory biomarkers in response to acute stress have been extensively studied, and have shown promise as an evaluation tool for determining stress levels and corresponding systemic inflammation [57]. In 2014, Cohen et al. performed a study in which salivary pH was measured as a biomarker of exam stress and a predictor of exam performance. The study consisted of 83 first and second-year university nursing students in two specific classes: microbiology for first years and pharmacology for second years. The exams for which saliva was evaluated were the students’ first term semester exams, and were considered very challenging and stressful due to a mandatory passing grade of 60 and 65 for microbiology and pharmacology, respectively. One hour before the exam (T1) students filled out a questionnaire and gave saliva samples. The second time point of collection (T2) was three months later, during a non-exam period, for which 68 students of the 83 participated. The pH of each saliva sample was taken immediately using a commercially available sensing device, the CyberScan pH 501, which is comprised of a kit of multipurpose sensors. The pH sensor connected to the device was dipped into the sample until a beep was heard, indicating the end of pH measurement [59]. 

Multiple regression analyses and standard statistical tests (*t*-tests and Pearson’s correlation) were then performed to assess correlations between study variables and pH, and pH and exam performance. Results indicated that salivary pH was higher at T2 (non-stressed) than T1 (prior to exam), and pH levels could successfully be predicted by the levels of appraised threat of the exam situation and the experienced stress. Emotionality of the stress exam predicted pH at the exam time only, as shown in Table 4 [59]. While this study only evaluated one biomarker for stress response, several studies have highlighted the potential of salivary metabolomics for stress evaluation and disease detection [57,59,60,61]. Evaluation of the whole saliva metabolome by e-tongue could potentially provide a rapid measurement of stress and anxiety, and these devices could be used as an aid in uncovering the underlying pathologies for a more targeted, personalized treatment. 

**Figure 18:**
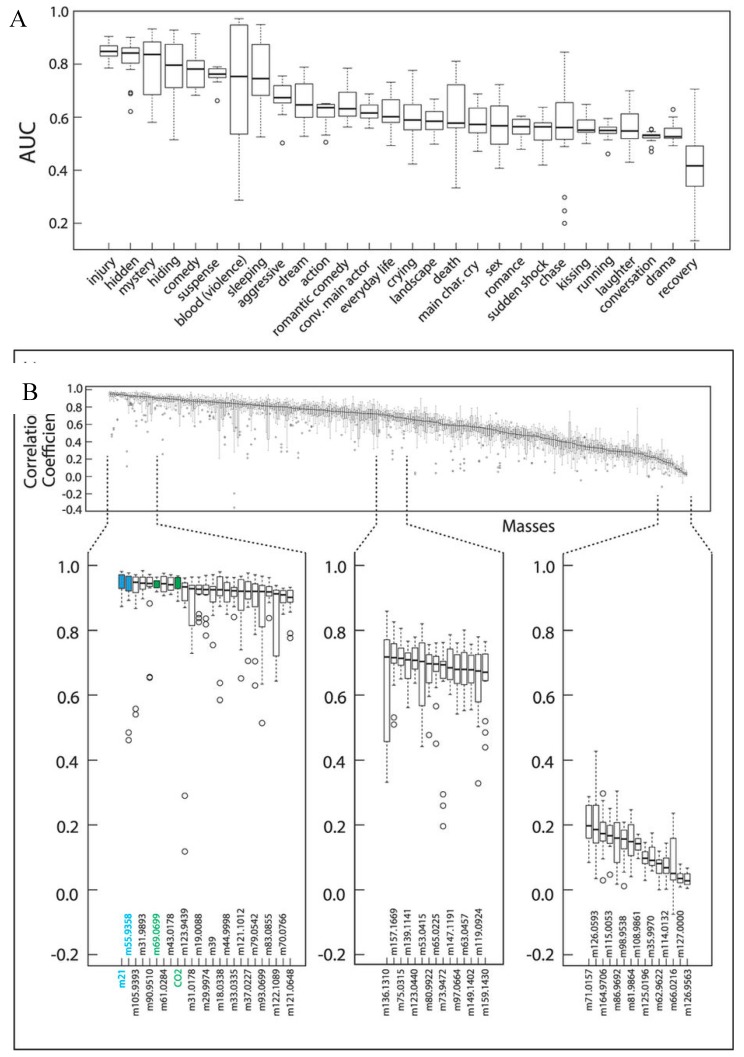
Shown are the results when two thirds of the whole film screening dataset is randomly selected (15 times) and the resultant model tested on the remaining third. The boxes indicate the extent of 25% of the data either side of the median (solid line). The dashed vertical line represents the lowest/highest datapoints that are still in the 1.5 interquartile range while the circles are outliers. (**A**) shows AUC which expresses the ratio between true positives (when the model correctly predicted labels based on mass decision trees) and false positives (backward prediction). A random prediction produces an AUC value of 0.5; (**B**) shows the ability of an individual mass to be predicted by the labels (forward prediction). The performance of this prediction versus the real value for VOC mixing ratios is given as the Pearson’s correlation coefficient (r). High correlation coefficients indicate the predictive model was successful for that particular species, and not that all species with high correlation coefficients are inter-correlated. Used with permission from [103].

**Figure 19:**
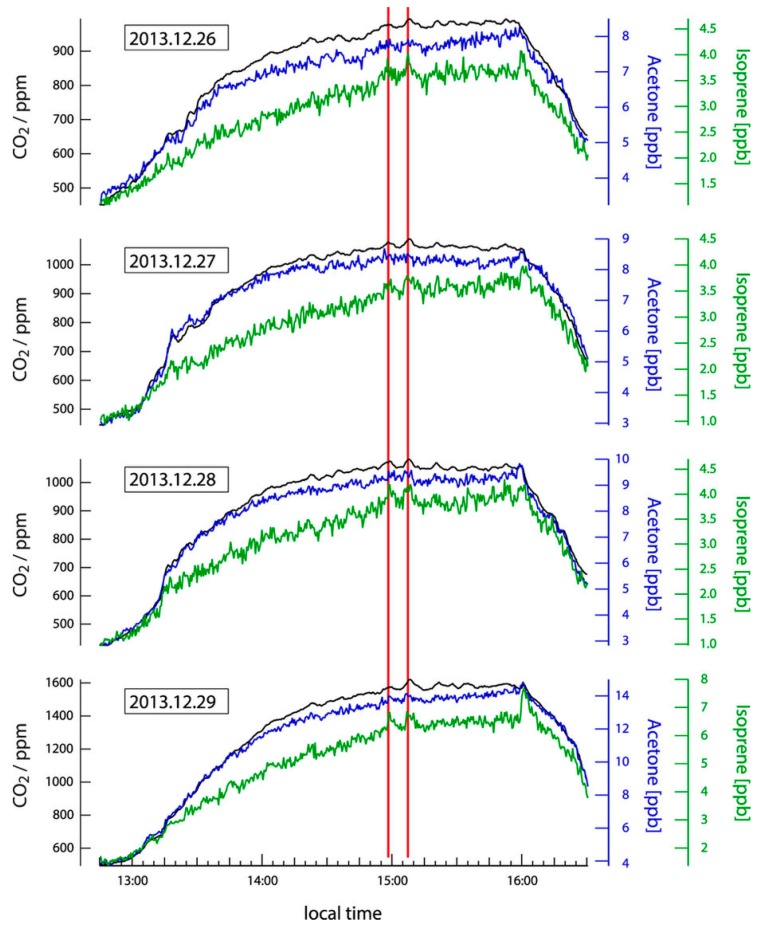
Measurements of CO_2_, isoprene and acetone taken during four separate screenings of “Hunger Games 2”. Used with permission from [103].

Though much less extensively studied than saliva, one study conducted by Williams et al. reported scene-specific emissions of VOCs from human subjects in cinema audiences. Scenes were categorized according to categories listed in the Internet Movie Database (IMDb). The study was conducted at the *Cinestar Cinema* in Mainz, Germany, and conducted in two separate screening rooms (capacity 230) between December of 2013 and January 2014 [103]. Measurements were taken from circulating air (cycled through six times per screening) by a proton transfer reaction time-of-flight mass spectrometer (PTR-ToFMS) [104] to measure carbon dioxide (CO_2_), isoprene, acetone, and other VOC concentrations. Overall, scenes labeled as “Injury” and “Comedy” had the highest overall causal link to measured species, shown by the AUC plot for predictive models in Figure 18, and both Isoprene and CO_2_ were predictors of scene intensity, as demonstrated in the plot for Hunger Games 2, as shown in Figure 19 [103]. 

## 6. Metabolic Disorders

### 6.1. Diabetes

As one of the first metabolic disorders to be evaluated through e-nose analysis of exhaled VOCs, much research has been performed to optimize a B-CRSA for the detection and monitoring of diabetes and comorbid disorders [31,105,106,107]. Diabetes Mellitus (DM) is divided into three categories: Type 1 (DMT1), characterized by absolute insulin deficiency [108], Type 2 (DMT2), characterized by high blood glucose in the context of insulin resistance and relative insulin deficiency [109], and gestational (GDM), characterized by insulin resistance during pregnancy [110]. It is widely known that all of the types of DM require close monitoring of glucose levels and personalized insulin dosage regimen for disease management and prevention of comorbid disorders and long-term impairment. While the breath metabolome of DMT1 and DMT2 have been evaluated [105,111,112] for diagnosis and glucose monitoring, the most recent studies have been focused on optimizing a device to elucidate the specific pathological processes of DM that influence comorbid disorders such as obesity, hyperlipidemia, coronary artery disease [113], and cognitive impairment [109].

To measure cognitive impairment influenced by DMT2, Mazzatenta et al. recorded breathprints of three DMT2 patients and three healthy controls while at rest and while performing a cognitive task in the form of Sudoku puzzles. Breath samples were obtained using similar methods to those described in previous sections, and breath signals were recorded for 30 s, 5 min prior to the test, while taking the test, and 5 min after the test was given. The B-CRSA employed in this study consisted of an iAQ-2000 that was equipped with a metal oxide semiconductor (MOS) sensor that changes resistance based on the number of VOCs adsorbed to the surface and provides a measure of VOCs as a ppm CO_2_ equivalents, denoted as VOCe. Upon breath exposure and measurement, VOCe amount was calculated by taking the integral of the signal curve within an established time point. Data from the sensor was normalized using the following equation:

(2) $$X=\frac{\left(x_{i}-x_{i,j,min}\right)}{\left(x_{i,j,max}-x_{i,j,min}\right)}$$

where “*X* is the normalized value obtained from the result of subtraction from a value × the minimal value in a series of values ranging from *i* to *j* divided by the result of subtraction from the maximal value in the same series the minimal value” [109]. 

**Figure 20:**
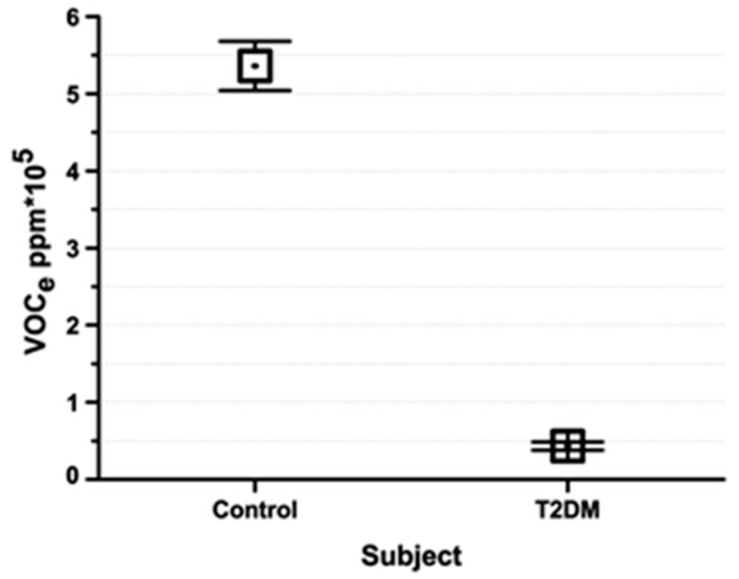
Comparison of the averaged volatile organic compounds (VOCs) as a ppm CO_2_ equivalents (VOCe), which is a measure of VOCs as ppm CO_2_ equivalents provided by the MOS sensor, in control vs. diabetic (T2DM) subjects. Measurements are from the 30-s pre-test interval. Used with permission from [109].

**Figure 21:**
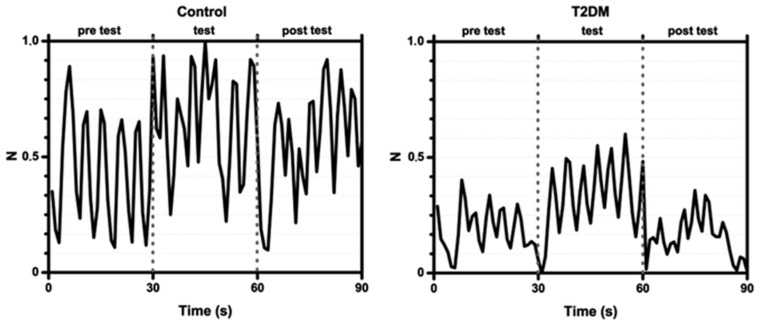
Normalized breath signal in a representative control subject compared with that in a diabetic (T2DM) subject, recorded through the consecutive phases of the experiment; pre-test: signal of normal free breathing, test: breathing signal during cognitive test; post-test: recovery breathing signal after the cognitive test. Normalization formula is *X* ¼ (*x_i_* − *x_i,_*_j_ *_min_*)/(*x_i,j max_* − *x_i, j min_*). Used with permission from [109].

For the VOCs picked up by the MOS sensor in both cases, the VOCe levels were predictably higher in the healthy control subjects prior to the test than that of the patients with DMT2, as shown in Figure 20. Overall, the breath profiles for both subject classes had the same shape (with a peak in VOCe levels during the cognitive task), though the mean VOCe levels for healthy controls were ten-fold higher than those of DMT2 for all three phases, as shown in Figure 21. This study aimed to gain insight into the effect of DMT2 on cognitive function, measureable from respective VOCe levels. Importantly, the study found that VOCe levels are significantly lower in DMT2 patients for both resting and testing phases as compared to healthy controls. The authors postulated that this could indicate impaired metabolic function caused by a resistance to insulin, carrying serious repercussions for cognitive memory and function, where insulin carriers and insulin receptors are critical components [109]. The impairment of metabolic processes in DMT2 has repercussions for many other systems in the body, and carries serious risk of the development of cardiovascular disease [113] and dementia later in life [109]. An e-nose device that is not only sensitive to DM-specific VOCs, but can also quantify VOCs in exhaled breath would be a highly beneficial tool for monitoring disease progression.

### 6.2. Inflammatory Bowel Disease (IBD) and Irritable Bowel Syndrome (IBS) 

IBD and IBS are both chronic conditions that affect the small and large intestines; however, important differences exist between the etiologies for each disease. IBD is an autoimmune disease with two main types: ulcerative colitis (UC), which affects the top layers of colonic mucosal tissue, and Crohn’s disease (CD), which may affect any part of the GI tract from the mouth to the anus, though it is usually confined to the end of the small intestine and the beginning of the colon [114]. While there are overlapping symptoms for UC and CD, it is important to distinguish between them to enact patient specific therapies, and to distinguish between active and remissive disease states [48]. To date, endoscopy is required for both the initial diagnosis of IBD and for disease monitoring over time, and there remains a need for a non-invasive diagnostic tool to reduce the significant burden on patients, both of the preparation process and for the procedure itself [51]. In contrast, IBS is a chronic functional disorder of the gastrointestinal system, the cause for which is largely unknown. To date, no biomarker has yet been found, and diagnosis is therefore based on reported symptoms of frequent abdominal pain and irregularity, requiring several weeks of symptom monitoring. As a result, there has been debate over the appropriate diagnostic criteria for IBS, resulting in a large margin of error for reported cases [115]. 

Fortunately, recent studies have demonstrated that both CD and UC are detectable through VOC analysis of either urine [48] or the fecal [51] headspace. It is widely known that IBD results in an alteration of the gut microbiome, and these bacteria cause a fermentation of the non-starch polysaccharides (fiber) ingested by the host [116]. The resultant products, also known as the fermentome, are detectable in both urine and fecal headspace, mainly due to the intestinal permeability associated with a disease flare-up [116]. In the most recent study with urine headspace, Arasaradnum et al. analyzed urine samples from 48 patients with IBD (24 with CD and UC, respectively) against 14 healthy control samples. A commercial device known as the Fox4000 (AlphaMOS, Toulouse, France) was chosen as the e-nose for this study. The Fox4000 B-CRSA consists of 18 metal oxide sensors, each of which responds to a complex VOC mixture with a unique resistance change. The collective sensor responses are then used to construct the smellprint of that sample. A field asymmetric ion mobility spectrometer (FAIMS) was used in conjunction with the e-nose to quantify and identify specific VOCs in urine samples as a validation of e-nose results. Further explanation is in Reference [48] but briefly, FAIMS separates chemical components of vapor on the principle of differences in ion mobilities within an electric field. Ion mobility is quantified after an asynchronous electric field is introduced to the ionized gas molecules, enabling chemical identity and classification.

E-nose data was then classified using PCA and discriminant function analysis (DFA) with the AlphaMOS software, and FAIMS data analyzed using Fisher’s Discriminant Analysis (FDA) with Matlab software. Overall, samples were separated into five classes: (1) CD no flare; (2) CD flare up; (3) UC no flare; (4) UC flare up; and, (5) healthy control. First, PCA results showed some separation between classes; however, because it is a non-classified technique, some overlap existed between classes. Upon processing with DFA, a pre-classified technique, obvious differences were observed for each sample class; moreover, accuracy of reclassification with five classes exceeded 70% (88% between purely disease classes and control, three classes). FDA with FAIMS data yielded similar results, with accuracy of reclassification between purely disease classes and control (3 classes) exceeding 75%, with 66% accuracy for distinguishing CD flare and quiescent state, and 74% accuracy for distinguishing UC flare and quiescent state [48]. The accuracy of the Fox4000 e-nose for detecting UC and CD, coupled with its ability to distinguish between disease states, shows much promise as a non-invasive diagnosis and disease-monitoring device for IBD. 

**Figure 22:**
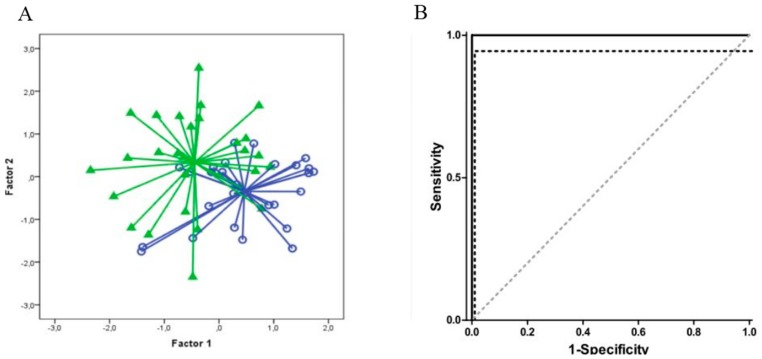
(**A**) Scatterplot for the discrimination of patients with ulcerative colitis at presentation (full triangles) and healthy controls (empty circles), by electronic nose. Axes depict two orthogonal linear recombinations of the original 32 sensor data by means of principal component analysis; (**B**) Receiver operator characteristic curve for the discrimination of ulcerative colitis and healthy controls at first presentation (solid line) and upon achieving remission (dashed line), by electronic nose. The areas under the curve ± 95% confidence interval (AUC ± 95% CI) with associated *p*-value were: first presentation 1.00 ± 0.00, <0.001; remission 0.94 ± 0.05, <0.001. Used with permission from [51].

**Figure 23:**
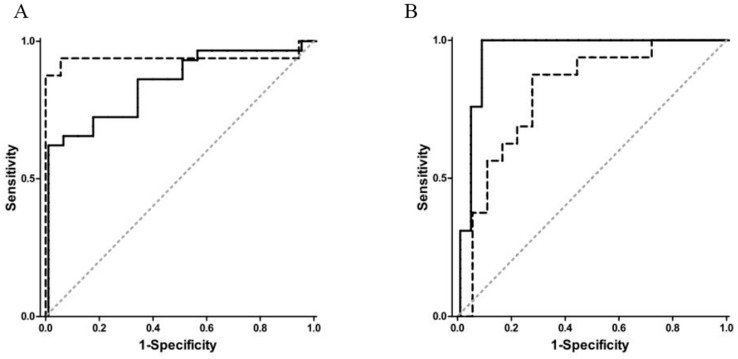
(**A**) Receiver operator characteristic curve for the discrimination of Crohn’s disease and healthy controls at first presentation (solid line) and upon achieving remission (dashed line), by electronic nose. The areas under the curve ± 95% confidence interval (AUC ± 95% CI) with associated *p*-value were: first presentation 0.85 ± 0.05, <0.001; remission 0.94 ± 0.06, <0.001; (**B**) Receiver operator characteristic curve for the discrimination of ulcerative colitis and Crohn’s disease at first presentation (solid line) and upon achieving remission (dashed line), by electronic nose. The areas under the curve ± 95% confidence interval (AUC ± 95% CI) with associated *p*-value were: first presentation 0.96 ± 0.03, <0.001; remission 0.81 ± 0.08, 0.004. Used with permission from [51].

As mentioned previously, IBD can also be detected through fecal headspace VOC analysis. One study conducted between April 2010 and January 2013 by de Meij et al. analyzed fecal headspace VOCs of 153 samples from 83 children (29 CD, 26 UC, and 28 age-matched controls). The Cyranose 320 was the e-nose of choice, and was exposed to heated samples via a closed-loop system, as described previously in Section 3.2. PCA was then performed on the Cyranose 320 data and predictive classification models constructed using CDA and validated by leave-one-out cross validation. An ROC was then plotted and the AUC calculated to evaluate accuracy. PCA plots and ROC curves for UC are shown in Figure 22. Overall, patients with UC could be successfully distinguished from healthy controls for both active cases (AUC ± 95% CI, *p*-value, sensitivity, specificity: 1.00 ± 0.00; *p* < 0.001, 100%, 100%) and those in clinical remission (0.94 ± 0.06; *p* < 0.001, 94%, 94%). Patients with CD could also be distinguished with confidence for both active cases (0.85 ± 0.05 *p* < 0.001, 86%, 67%) and those in clinical remission (0.94 ± 0.06 *p* < 0.001, 94%, 94%). Moreover, patients with UC differed from patients with CD for both active and remissive cases with excellent confidence (Active: 0.96 ± 0.03; *p* < 0.001, 97%, 92%; Remissive: 0.81 ± 0.08, *p* = 0.004, 88%, 72%), as shown in Figure 23. This demonstrates that fecal headspace analysis by the Cyranose 320 is a feasible method for both IBD diagnosis and monitoring [51]. 

In addition to urine and fecal headspace analysis for IBD detection, alterations in the gut microbiome associated with IBD have also been shown to alter VOC profiles of exhaled breath [39,117]. One study employed selected ion flow tube mass spectrometry (SIFT-MS) as a VOC analysis method, and models were constructed from the data using partial least squares discriminant analysis with orthogonal signal correction (OSC PLS-DA) to successfully distinguish between UC patients, CD patients, and healthy controls with a good sensitivity and specificity [39]. While this was a good pilot study to elucidate VOCs of interest, SIFT-MS is a specialized and costly analysis method that requires technician expertise, and it can only detect VOCs that can be ionized by preselected precursor ions [118]. Thus, SIFT-MS is impractical for widespread use as a non-invasive diagnosis method for IBD. Instead, future studies should be directed toward employing e-nose devices that contain B-CRSAs that are optimized to detect IBD-specific VOCs, as previously determined by MS analysis. 

In 2013, Gao et al. designed a virtual e-nose to detect H_2_, CH_4_, and CO_2_ in breath for the detection of small intestinal bacterial overgrowth (SIBO) as a diagnostic etiological tool for IBS [117]. SIBO is bacterial overgrowth with upper respiratory tract flora and with Gram-negative bacteria, caused by failure of the gastric acid barrier and nutrient malabsorption in the upper intestine. Detection and evaluation of severity require aspiration and direct culture of the jejunal contents; however, this method is invasive, has low reproducibility, and is only able to detect the presence of bacteria that can be cultured (<50%) [119]. Therefore, breath analysis has been employed as an alternative analysis method to detect gases, including H_2_ and CH_4_ from bacterial fermentation of poorly absorbed carbohydrates, such as glucose, with promising results [120]. The virtual e-nose in the present study used gas chromatography (GC) to filter breath samples, retaining H_2_ and CH_4_ for analysis. Briefly, the sample is pumped into a sampling loop after filtering out water, and H_2_ and CH_4_ are separated from breath samples using GC columns before being passed over a MOS sensor. 

The output response of the MOS sensor (represented by plotting the change in electrical potential vs. time) contains two distinct peaks, the first corresponding to H_2_ concentration and the second corresponding to CH_4_ in ppm. Results indicated that the sensor had a detection range of 1–550 ppm for both gases. Diagnostic modeling was performed for the device using samples from local patients. All of the subjects ingested a challenge dose of carbohydrates (e.g., lactose), which would cause the H_2_ and CH_4_ levels to rise significantly in breath within one to two hours, only if the sugar is not digested and reaches the colon. Concentrations of both H_2_ and CH_4_ for the control groups were much lower than those with SIBO [117]. This virtual e-nose system is small and non-invasive, and has shown excellent sensitivity to H_2_ and CH_4_ with good reproducibility. While this device has shown potential for the detection of VOCs associated with SIBO, results were only recently presented in the 2017 ISOCS/IEEE International Symposium on Olfaction and Electronic Nose, and larger scale studies are needed to validate the accuracy and feasibility of the device. 

### 6.3. Chronic Kidney Disease (CKD)

Globally, CKD has the 18th highest mortality rate, and is the third highest in the increase of number of years of life lost due to disease from 1990 to 2010 [121]. CKD also carries a risk of comorbid disorders, such as cardiovascular disease, that can further decrease quality and longevity of life [121,122]. Similar to IBD, CKD is a metabolic disorder that has a marked impact on the gut microbiome, causing inflammation, oxidative stress, and impaired digestive function [123]. Impaired renal clearance of nitrogenous wastes, including urea, results in a passive diffusion and/or secretion into the gastrointestinal tract. This waste is then fermented by the gut bacteria, and leads to emission of VOCs, detectable in both fecal headspace and exhaled breath [50]. Indeed, many studies have identified ammonia in exhaled breath as a biomarker for CKD detection and disease state [124]. Currently, the gold standard of CKD diagnosis is a calculation of glomerular filtration rate (GFR) and measurement of albuminuria; however, GFR must be estimated by measuring the creatinine concentration in plasma, which can be inaccurate as creatinine concentration is influenced by several other factors [121]. Hence, there remains a need for a non-invasive, accurate method to identify and monitor CKD.

**Figure 24:**
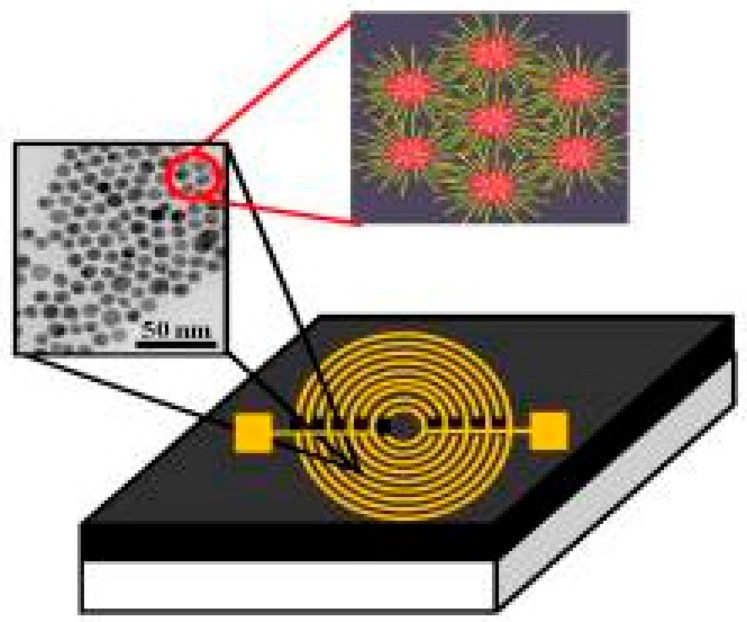
Schematic representation of the gold nanoparticle (GNP) sensors in this study (not drawn to scale). The sensors were formed by successively drop casting the solutions of the molecularly modified GNP solutions onto 10 pairs of pre-prepared Ti/Au-inter-digitated electrodes. The left inset in the sensor’s schematics shows a tunnelling electron micrograph (TEM) of the NPs, which connects the electrodes and forms multiple paths between them. The right inset of the sensor’s schematics shows schematics of films based on molecularly modified GNPs. In these films, the metallic particles provide the electric conductivity, and the organic film component provides sites for the sorption of analyte (guest) molecules. In addition to their role as an adsorptive phase, the presence of well- defined organic spacers (i.e., capping molecules) allows a control over the inter-particle distance, and thereby, obtaining nearly uniform inter-particle distances in the composite films. This allows achieving controlled signal and noise levels. Used with permission from [125].

In 2012, Marom et al. conducted a study to detect and monitor CKD conditions using an B-CRSA of gold nanoparticle (GNP) sensors, previously successful at detecting colorectal, lung, breast, and prostate cancer from exhaled breath analysis [125]. The study contained 17 patients with intermediate CKD (stages 2 and 3), 20 patients with advanced CKD (stages 4 and 5), and healthy controls. Subjects were aged 22–83 years, and patients were staged according the estimated GFR from plasma creatinine levels. Breath samples were collected in a similar manner to that described in previous sections and fully outlined in [126], where VOCs from ambient air were filtered out and only exhaled alveolar air was collected for analysis. Each of the sensors in the B-CRSA device was made up of 10 pairs of circular interdigitated gold electrodes, 3 mm in diameter, imbibed with GNPs that had been previously functionalized with organic ligands, as shown in Figure 24 [127]. The electrodes operate on the principle of chemiresistance, wherein a change in electrode resistance occurs upon analyte binding, measureable as a function of time by an Agilent Multifunctional switch. In all, there were 20 different uniquely functionalized sensors available, and the array was optimized for CKD detection in breath by selecting the four types of sensors that represented the most distinct and reproducible response upon calibration with clinically relevant, synthetic VOC vapors, and multivariate classification techniques [125].

The sampling system delivers pulses of breath to the enclosed sensor chamber, and exposure response is recorded over a period of 5 min, repeated two to three times to test reproducibility [125]. To measure time-dependent sensor response, four data points were collected for each sensor (S): (F1) resistance upon exposure, (F2) AUC of the response curve, (F3) sensor response time, and (F4) relaxation time at the end of exposure. CKD-specific response fingerprints were then determined using a supervised learning and classification method known as support vector machine (SVM) [128], which aims to find the best separation line between two data sets made up of the collective sensing signals, and automatically choose the most descriptive and suitable set of sensing features (i.e., best types of functionalized sensors to represent CKD breath). PCA was then applied to SVM data and plotted to show representation in three-dimensional principle component space. Cross-validation was performed to evaluate sensitivity and specificity.

SVM results suggested that breath from CKD patients in stages 2 and 3 was best represented by feature F1 of sensor S1, and three independent sensing features of sensor S2 (F1, F2, and F4). From this data, SVM results produced 77% sensitivity, 80% specificity, and 79% accuracy for detecting early stage CKD. For the monitoring of disease progression for patients from early to late stage and between stages 4 and 5 (typically when dialysis treatment begins), descriptive sensing features were also determined using SVM. From early to late stage, only a single sensing feature from sensor S1 was needed for accurate classification, producing a sensitivity, specificity, and accuracy of 75, 77, and 76%, respectively. For progression from stage 4 to stage 5, a suitable set of five sensing features was selected from SVM results S1, S3, and S4: specifically, F2–F4 of S1, F2 of S3, F1 of S4, producing a sensitivity (75%), specificity (92%), and accuracy (85%). 

### 6.4. Future Direction: Plasma Lipid Measurement through Exhaled Breath 

In addition to the commonly measured insulin and blood glucose levels, the measurement of plasma lipids in patients with diabetes offers significant benefits for the prevention of cardiovascular disease, longevity, and the improvement of quality of life. As with most diabetes monitoring, plasma lipid levels are most commonly measured through blood based assays; however, Minh Tdo et al. proposed that plasma lipids, specifically triglycerides (TG) and free fatty acids (FFA), could be successfully measured through exhaled VOC profiling. They hypothesized that “by integrating measurements of multiple exhaled VOCs at several consecutive time points, it is possible to estimate plasma concentrations of a given variable through multivariate regression analysis” [113]. In this study, 23 healthy volunteers were induced with hyperglycemia or hyperlipidemia through an IV infusion of insulin, glucose, and lipids in the antecubital vein to avoid any confounding effects from metabolism and absorption in the GI tract. Blood assays were also performed to measure the correlation between breath profiles and blood lipid and glucose levels. Blood, breath, and room air samples were collected at 12 time points over a period of 4 h to monitor subsequent changes. 

Breath was analyzed by an analytical instrument comprised of three gas chromatographs (GCs), which used different combinations of electron-capture detectors, flame-ionization detectors (FIDs), sulfur chemiluminescence detector (SCD), and a quadrupole mass spectrometer detector (MSD). Each of these detectors is sensitive to different types of VOCs, and the recorded data is combined to construct a comprehensive, quantitative VOC profile. Sample flow was then split between detectors, and data for each detector recorded by machine software. All of the VOCs are then quantified by integrating the area under each peak of the chromatogram. Prediction models built from the chromatograms were highly accurate with an *r* value of 0.97 and 0.90 for TG and FFA levels, respectively [113].

## 7. Future Directions and Remaining Challenges for B-CRSA Diagnostics

### 7.1. Remaining Technological Challenges

Though gravimetric and electrical sensors have been proven to be successful, there are many limitations with this device setup. Inaccuracies due to subtle changes in surface coating, humidity, or temperature necessitate frequent calibrations. The setup, preparation, and calibration process is unfortunately delicate and time-consuming [67,129]. B-CRSA systems involving optical sensors have shown much promise as they provide a more facile and cost-effective way of identifying analytes while maintaining accuracy. Optical sensors offer significant benefits when compared to those mentioned above since they can provide multiple complex data types simultaneously, including changes in intensity, fluorescence lifetime, wavelength, and spectral shape [130]. This approach increases the ratio of recognizable analytes to number of sensors used.

### 7.2. New Technological Improvements

Though accurate and able to differentiate between several pathologies, the Cyranose 320 is financially out of reach for the general consumer with a cost of *ca*. $8000, and requires specialized training, software, and nanosensor chips for each sample. Within the past five years, researchers have developed new B-CRSAs that take advantage of current technological advances, including functionalized carbon nanotube field effect transistor (FET) sensors, and metal oxide semiconductor (MOS) sensors, proposing devices with improved sensitivity, selectivity, and stability. With the development of more extensive and selective sensing mechanisms alongside advances in electronics and signal processing, B-CRSA devices have become smaller, more selective, and more sensitive, with fast data processing time and facile readout of vapor analysis [54,66,131,132,133,134].

Recent advances in science and technology have progressed toward personalized medicine, where large amounts of data can be analyzed to identify specific biomarkers of a disease in each individual patient. B-CRSA technology specifically provides a significant contribution to the personalized medicine approach and recent technological advances have produced devices with higher disease specificity, sensitivity, and ease-of-use.

### 7.3. Sampling

As e-nose and e-tongue device implementation continues to grow in breadth, there are certain limiting factors that must be addressed. For exhaled breath, following the capnogram cycle, which is a measure of inhaled and exhaled carbon dioxide concentration, exhalation is composed of three phases, the second of which, alveolar air, contains the VOCs of interest in disease diagnosis. The capnogram cycle shows that the composition of exhaled air greatly varies between stages of exhale and breathing velocity affects the rate of mixing between dead space air (phase one) and alveolar air [106]. Breath collection optimization may be a difficult goal to realize when characterizing diseases that affect patients’ breathing rate and forced vital capacity, such as COPD [90]. When developing a vapor sampling method, it is important to optimize the collection method, minimizing VOC interference from ambient air while capturing air that contains the highest concentrations of VOCs from the patient. VOC recovery is also affected by the sample storage material and time of storage [32]. 

While liquid sampling is more straightforward, variance in sample collection methods including sample storage, dilution/concentration, flow rate, and introduction to the array could still influence the accuracy of detection and cause heterogeneous results between studies. In addition, precautions need to be taken to ensure that the sample is not affected by patient factors, such as food and drink ingested before sampling, medications, and smoking habits. Even after obtaining an ideal sample, e-nose, and e-tongue performance accuracy may be limited by extrinsic factors, such as humidity and temperature, and intrinsic factors, such as sensor drift and instrumentation errors. Additionally, e-nose and e-tongue fingerprint analysis via pattern recognition requires complex data analysis, which currently limits the widespread implementation of these devices. 

Overall, B-CRSA device development and implementation would benefit greatly from an accepted standard for device performance evaluation and sample collection. While preliminary studies have been largely successful, rate of reproducibility is limited because methods must be optimized *de novo* for each specific application. Standards need to be developed from statistical analysis of device performance and should include thresholds for success in areas, such as response reproducibility and disease specificity and sensitivity. In developing these standards, it is also important to consider the ultimate goal of the device. For example, if the goal is simply to diagnose and classify a disease, selectivity is more important than sensitivity; however, if the goal is to monitor disease progression, sensitivity to slight variations in VOC profiles is of great importance.

## Figures and Tables

**Table 1:** Accuracy of Statistically Validated Breath Biosignature Models. Used with permission from [74].

Groups Compared (*n*)	Model 1	Model 2	Model 3	Model 4
Non-small cell (83) Controls (137)	0.701	0.811	0.761	0.710
Adenocarcinoma (50) Controls (137)	0.784	0.747	0.825	0.695
Squamous cell (23) Controls (137)	0.708	0.841	0.849	0.768
Adenocarcinoma (50) Squamous cell (22)	0.889	0.742	0.864	0.517
Small cell (9) Controls (137)	0.800	0.824	0.890	0.763
Small cell (9) Non-small cell (83)	0.752	0.752	0.781	0.584
Stages I and II (41) Stages III and IV (42)	0.792	0.793	0.784	0.460
Survival Survival <12 mo (24) >12 mo (68)	0.768	0.761	0.770	0.576

The groups compared for each question are listed. This is followed by the C-statistic (area under the receiver operating characteristic curve) for the statistically validated models (model 1 = selected sensor parameters only, model 2 = selected sensor and selected clinical parameters, model 3 = selected sensor parameters and all four clinical parameters, model 4 = all four clinical parameters only).

**Table 2:** Receiver operating characteristics analyses of breath-prints between colonized COPD patients, non-colonized COPD patients and healthy controls. Used with permission from [88].

	Colonized vs. Non-Colonized COPD Patients	Colonized COPD Patients vs. Healthy Controls	Non-colonized COPD Patients vs. Healthy Controls
Cross-validation accuracy	89%	88%	83%
Sensitivity	0.82	0.80	0.81
Specificity	0.96	0.93	0.86
AUROC	0.922	0.986	0.937
Positive predictive value	0.87	0.89	0.92
Negative predictive value	0.92	0.87	0.72
AUROC: area under the receiver operating characteristic.			

**Table 3:** Base materials and organic functionalities of the nanomaterial-based sensors. Used with permission from [37].

Base Material	Sensor No.	Organic Functionality	DFA Model 1 ^1^	DFA Model 2 ^2^	DFA Model 3 ^3^	DFA Model 4 ^4^
RN-CNTs	1	α-CD		X		
	1	β-CD		X		
	2	Carboxy-methylated β-CD				X
	3	Hydroxypropyl-β-CD	X		X	X
	4	Heptakis(2,3,6-tri-O-methyl)-β-CD		X		X
GNPs	5	2-mercapto-benzoxazole				X
	6	3-mercapto-propionate	X		X	X

The sensors that were selected for the four DFA models are marked with X. ^1^ Distinction of Alzheimer’s disease (AD; target group) from healthy (control group); ^2^ Distinction of Parkinson’s disease (PD; target group) from healthy (control group); ^3^ Distinction of AD (target group) from PD (control group); ^4^ Distinction between AD, PD, and healthy.CD: cyclodextrin; DFA: Discriminant factor analysis; GNP: Gold nanoparticle; RN-CNT: Random network of carbon nanotubes

**Table 4:** Means (SDs) and range of study variables for Time 1 (T1) and Time 2 (T2). Used with permission from **[59]**.

	T1		T2		
	M	SD	M	SD	*t*(67)
Threat appraisal	5.93	1.56	4.70	1.61	5.55 **
Challenge appraisal	7.10	1.12	6.95	0.89	0.98
Experienced stress	6.01	1.89	5.72	1.27	2.05 *
Worry	2.28	0.70	2.18	0.75	1.17
Emotionality	2.59	0.68	2.45	0.74	1.70
Text anxiety (total score)	2.53	0.59	2.32	0.63	2.50 *
pH	6.95	0.60	7.41	0.74	−4.33 **
Test performance	69.33	14.11			

* *p* < 0.05; ** *p* < 0.001*.*
